# Supplementary material for: Effective high-throughput RT-qPCR screening for SARS-CoV-2 infections in children
Source: Nat Commun. 2022 Jun 25;13:3640. doi: 10.1038/s41467-022-30664-2 (PMC9233713; doi:10.1038/s41467-022-30664-2)
Supplement: Supplementary file 1 — Supplementary Information [file 41467_2022_30664_MOESM1_ESM.pdf]

Supplementary Information

for

Effective high-throughput RT-qPCR screening for SARS-CoV-2 infections in children

Supplementary Figures

**Supplementary Figure 1**	Cohort of acutely infected individuals
**Supplementary Figure 2**	Pool-RT-qPCRs and testing areas of 12 laboratories
**Supplementary Figure 3**	Data collection and plausibility checks
**Supplementary Figure 4**	Turn-around time of RT-qPCRs
**Supplementary Figure 5**	Implementation of the Lolli-Method in daycare facilities
**Supplementary Figure 6**	Number of infected individuals in positive pool-RT-qPCRs
**Supplementary Figure 7**	Determination of the SARS-CoV-2 detection rate of the Lolli-Method
**Supplementary Figure 8**	Correlation between Ct-values of pool-RT-qPCR and matched single-RT-qPCRs
**Supplementary Figure 9**	Modelling differences in infections dynamics of SARS-CoV-2 variants with the Lolli-Method in schools

Supplementary Tables

**Supplementary Table 1**	SEIR-model parameter description
**Supplementary Table 2**	Tested schools and students
**Supplementary Table 3**	Equipment for SARS-CoV-2 screening in schools

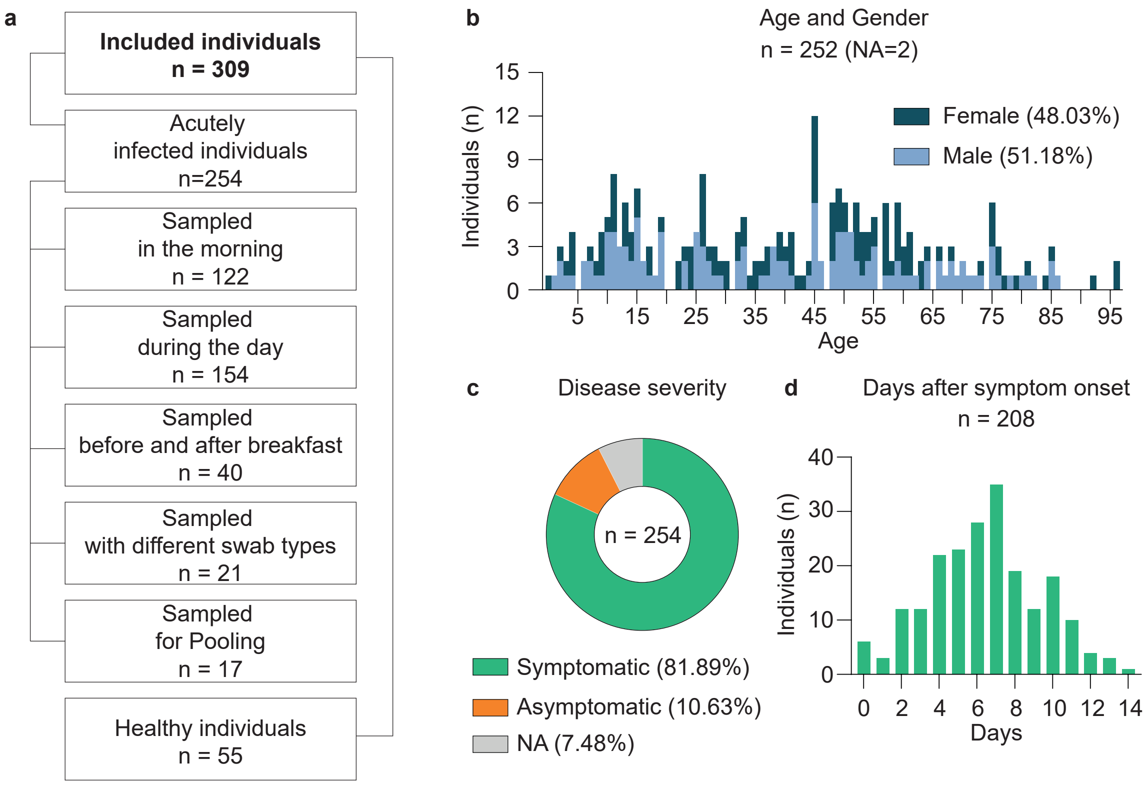

Supplementary Fig. 1: Cohort of acutely infected individuals
a, Flowchart including cohort sizes selected for RT-qPCR for the validation of the Lolli-Method. **b**, Age and gender distribution of the cohort. **c**, Disease severity among participants. **d**, Days after symptom onset among participants.

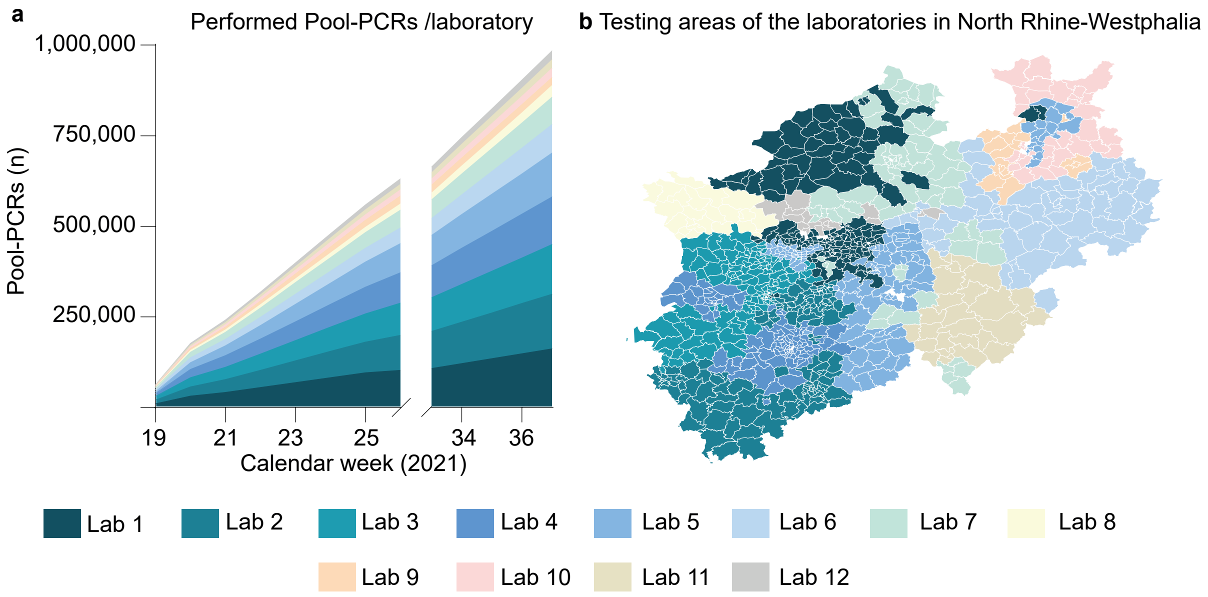

Supplementary Fig. 2: Pool-RT-qPCRs and testing areas of 12 laboratories
a, Cumulative number of performed pool-RT-qPCRs for each laboratory stratified by calendar week. **b**, Testing areas in North Rhine-Westphalia of each laboratory. Each laboratory is assigned one corresponding color.

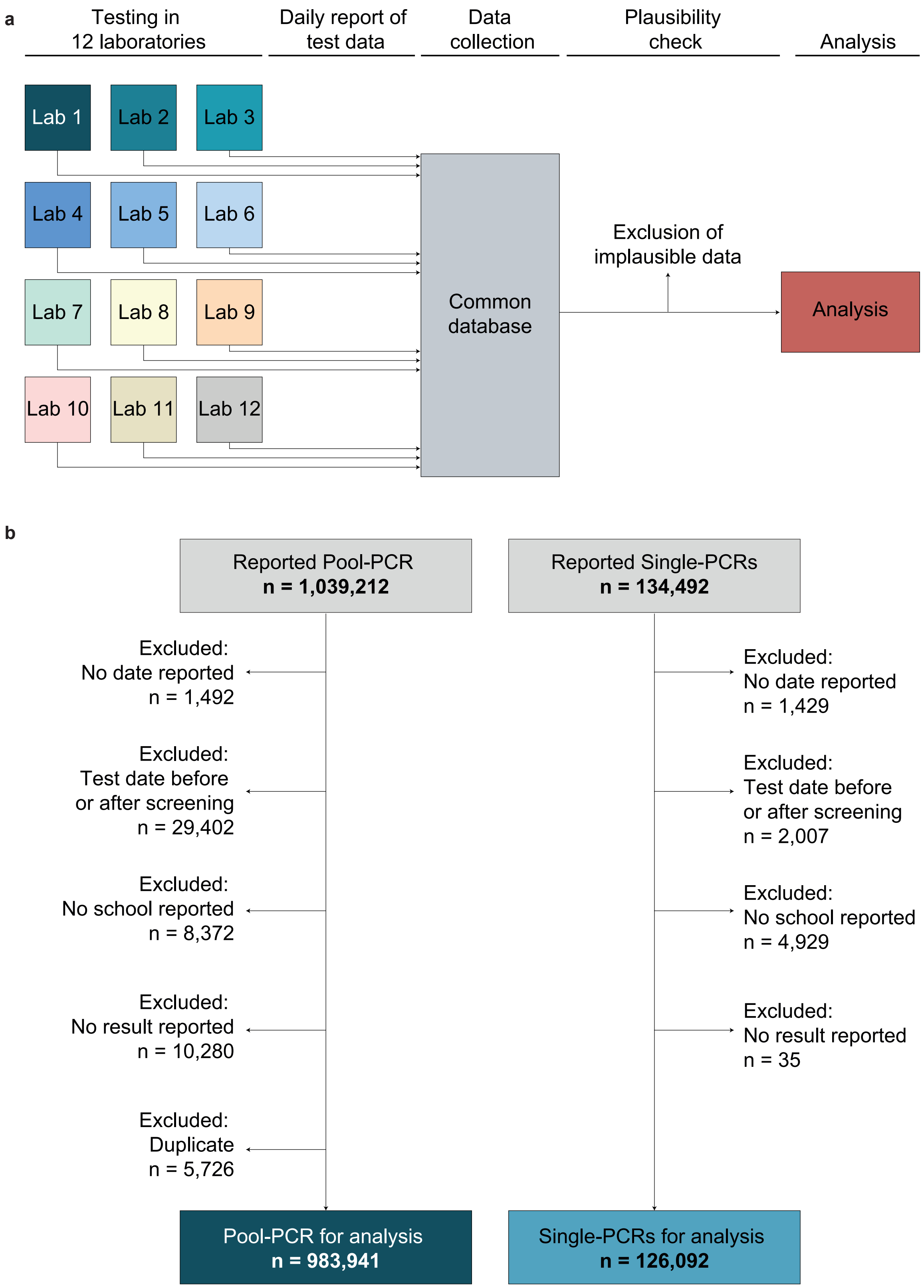

Supplementary Fig. 3: Data collection and plausibility checks
a, Flowchart including steps of data collection and quality control. **b**, Flowchart including details of data plausibility checks for RT-qPCRs indicating the numbers of RT-qPCRs that were used for analysis.

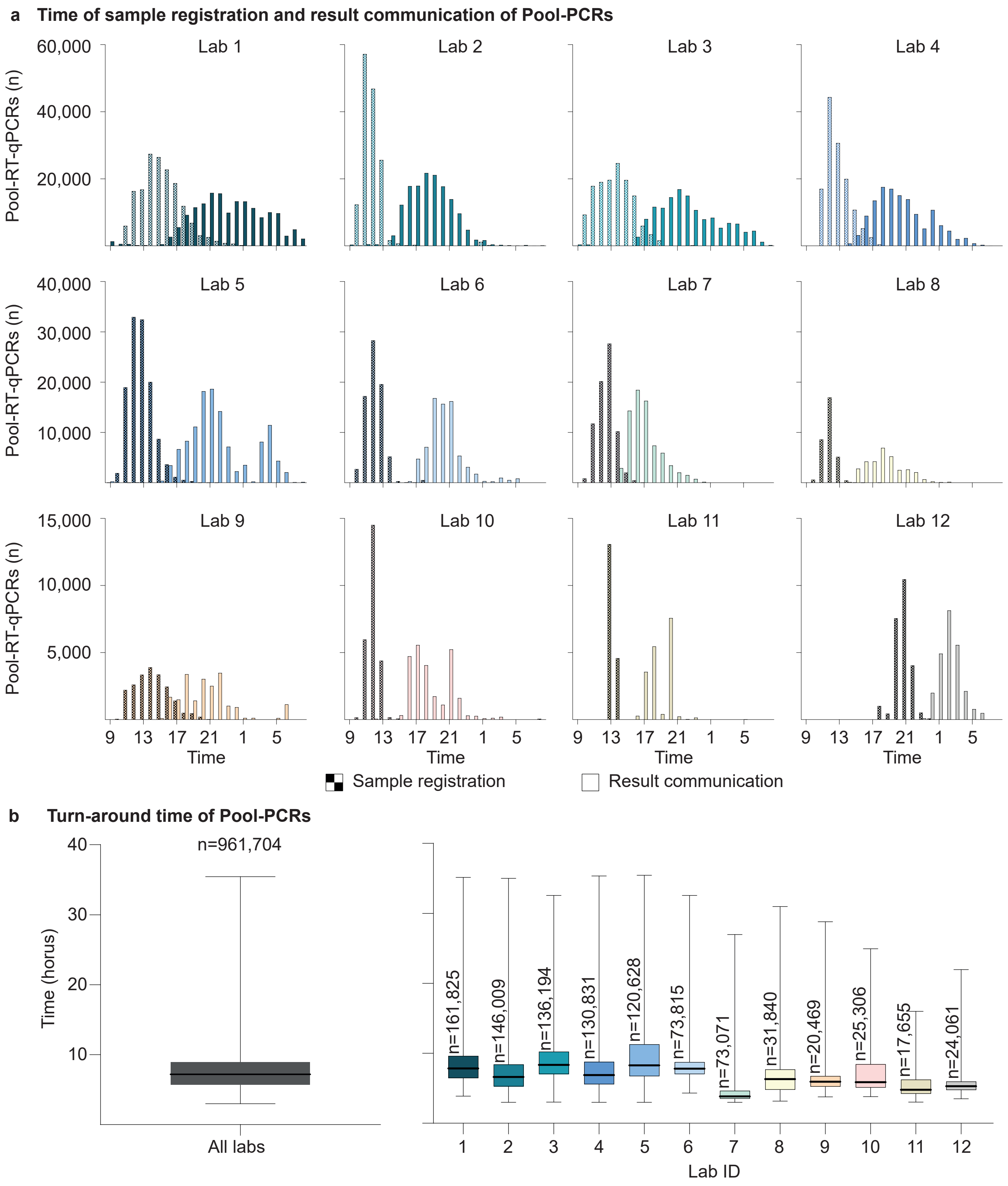
a Time of sample registration and result communication of Pool-PCRs
Pool-RT-qPCRs (n)
60,000
40,000
20,000
Lab 1
Lab 2
Lab 3
Lab 4
40,000
30,000
20,000
10,000
Lab 5
Lab 6
Lab 7
Lab 8
15,000
10,000
5,000
Lab 9
Lab 10
Lab 11
Lab 12
9 13 17 21 1 5
Time
Sample registration
Result communication
b Turn-around time of Pool-PCRs
Time (horus)
40
30
20
10
n=961,704
All labs
n=161,825
n=146,009
n=136,194
n=130,831
n=120,628
n=73,815
n=73,071
n=31,840
n=20,469
n=25,306
n=17,655
n=24,061
1 2 3 4 5 6 7 8 9 10 11 12
Lab ID

c Time of sample registration and result communication of Single-PCRs

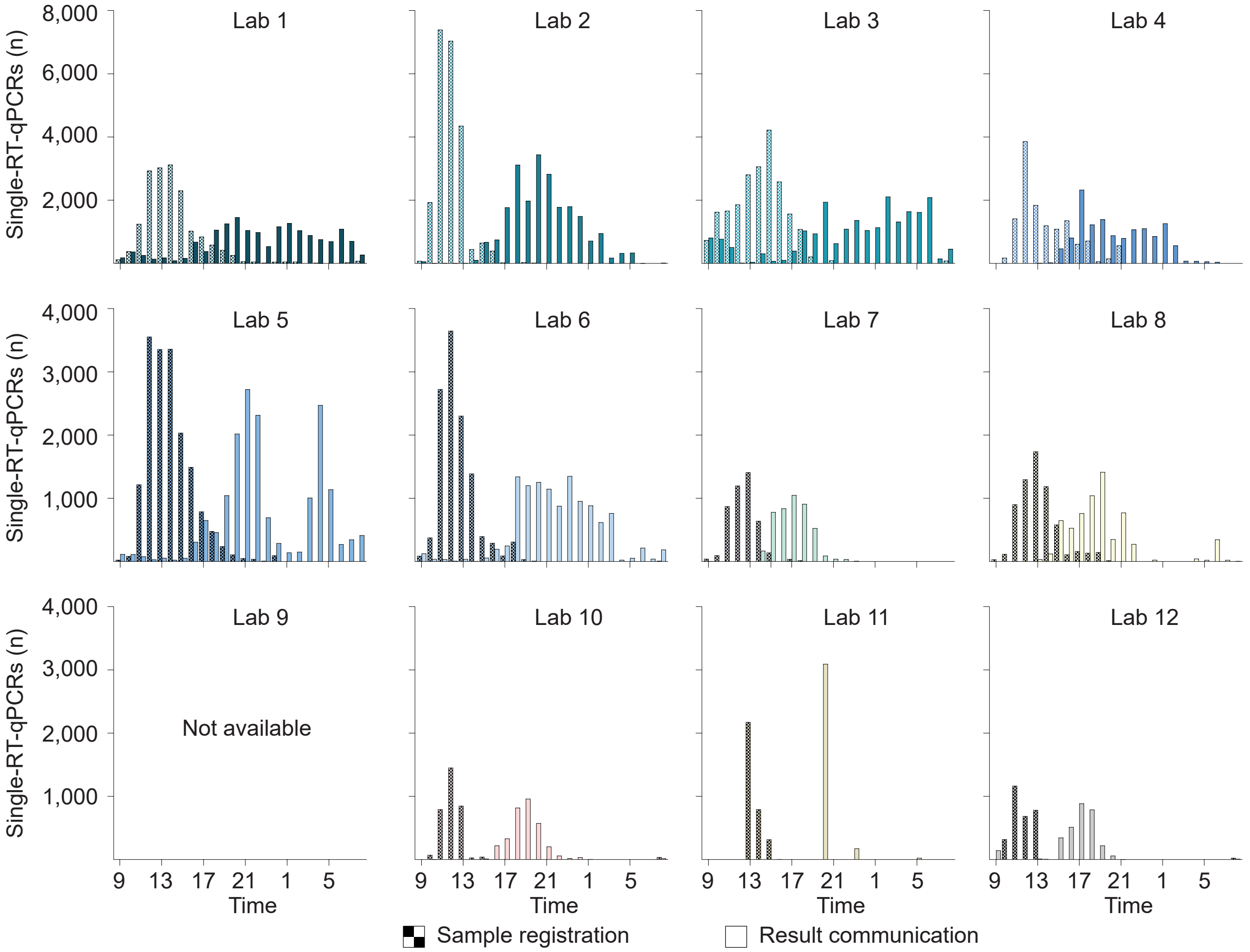

d Turn-around time of Single-PCRs

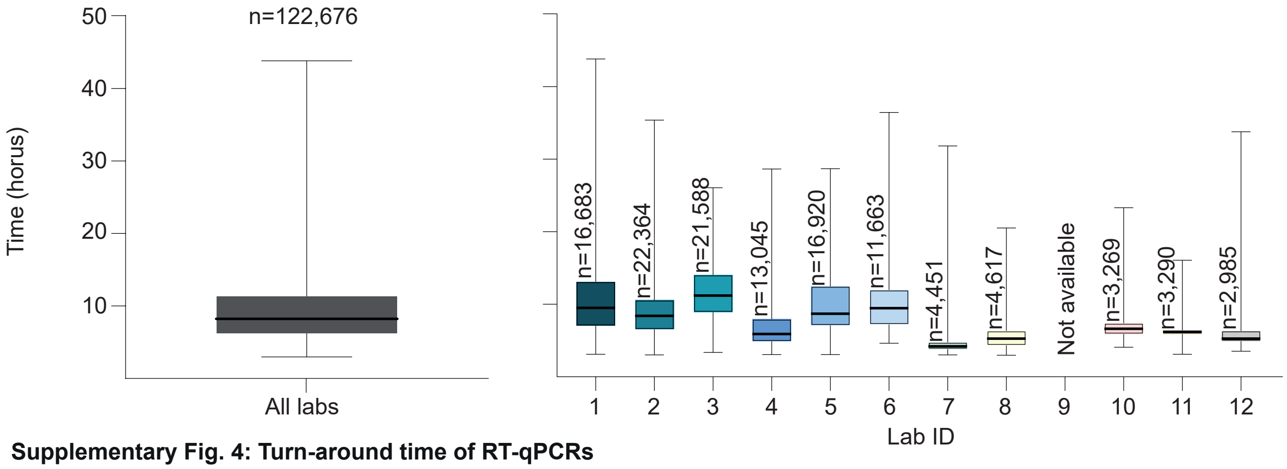

Supplementary Fig. 4: Turn-around time of RT-qPCRs
a, The bar charts represent the time of registration and result communication of pool-RT-qPCRs of each laboratory. **b**, Turn-around time of pool-RT-qPCRs (n=961,704 independent pool-RT-qPCRs). The horizontal lines in the green Box-Whisker-Plot indicate the medians, the lines at the top and at the bottom of the boxes indicate first and third quartiles and the error bars represent minimum and maximum turn-around times **c**, The bar charts represent the time of registration and result communication of single-RT-qPCRs of each laboratory. **d**, Turn-around time of single-RT-qPCRs (n=122,676 independent single-RT-qPCRs). The horizontal lines in the green Box-Whisker-Plot indicate the medians, the lines at the top and at the bottom of the boxes indicate first and third quartiles and the error bars represent minimum and maximum turn-around times.

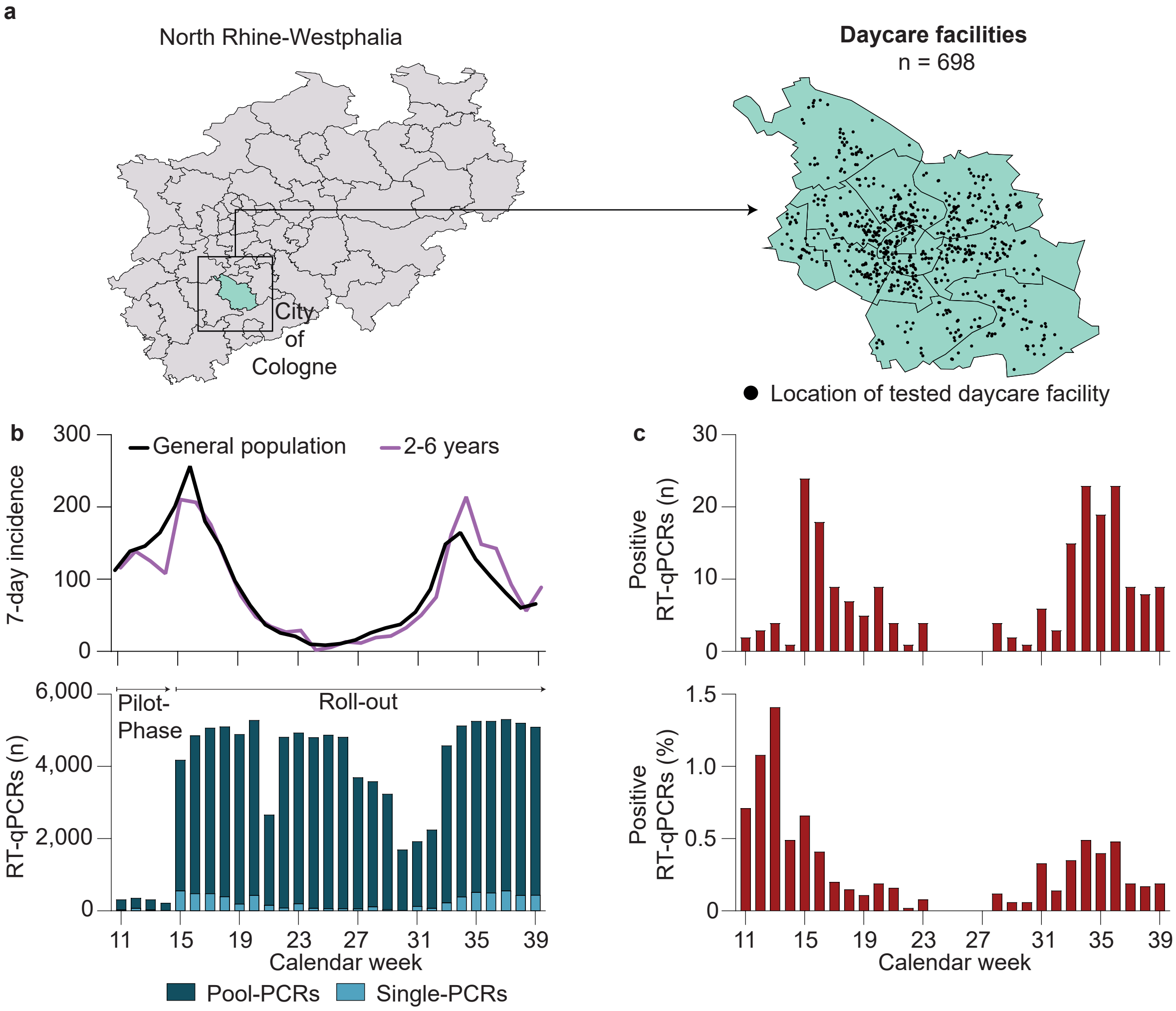

Supplementary Fig. 5: Implementation of the Lolli-Method in daycare facilities

a, Map of North Rhine-Westphalia indicating the locations of the tested daycare facilities in the city Cologne. **b**, SARS-CoV-2 7-day incidence in Cologne (top) and number of performed RT-qPCRs (bottom) stratified by calendar week. **c**, Number of positive pool-RT-qPCRs and rate of positivity of pool-RT-qPCRs stratified by calendar week.

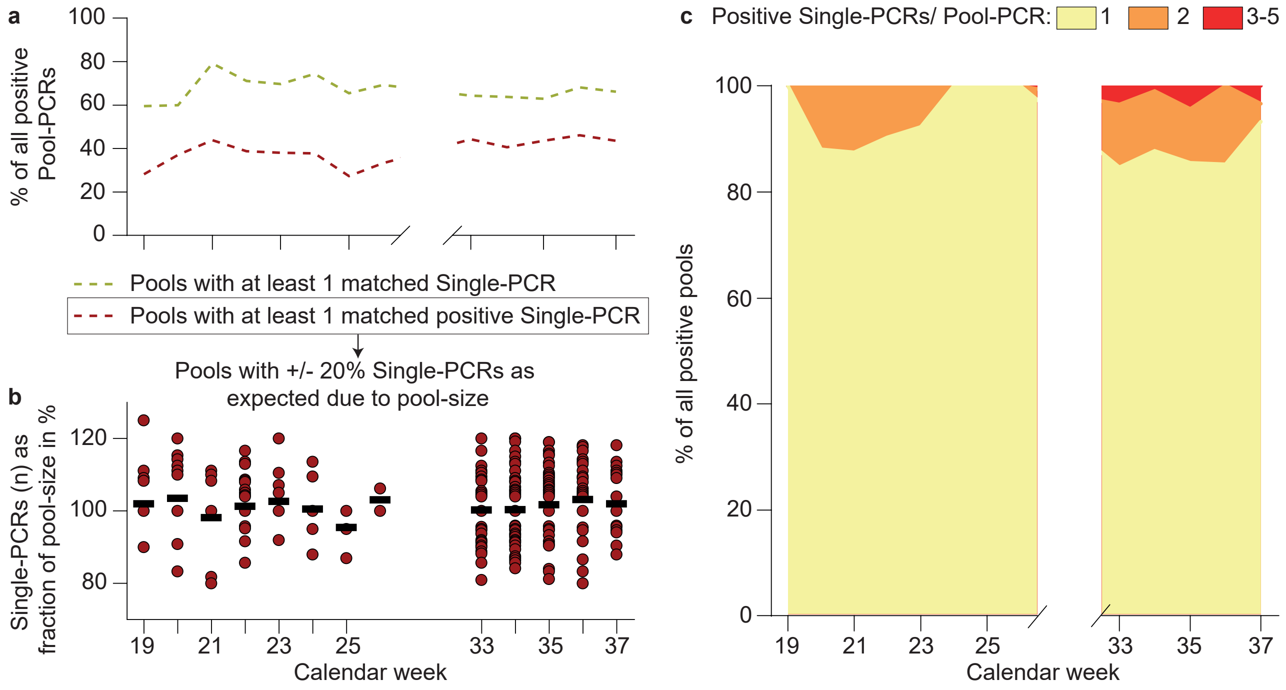

Supplementary Fig. 6: Number of infected individuals in positive pool-RT-qPCRs

a, Overview on data quality stratified by calendar week. **b**, Only pool-RT-qPCRs with at least one assigned positive single-RT-qPCR were used for further analysis. Out of those, only pool-RT-qPCRs were analyzed when the number of single-RT-qPCR was between 80 and 120% of the expected number, according to the reported pool-size (n=470 independent pool-RT-qPCRs). Horizontal lines represent the means of the fractions stratified by calendar week. **c**, Categorization of number of positive single-RT-qPCRs/positive pool-RT-qPCR stratified by calendar week.

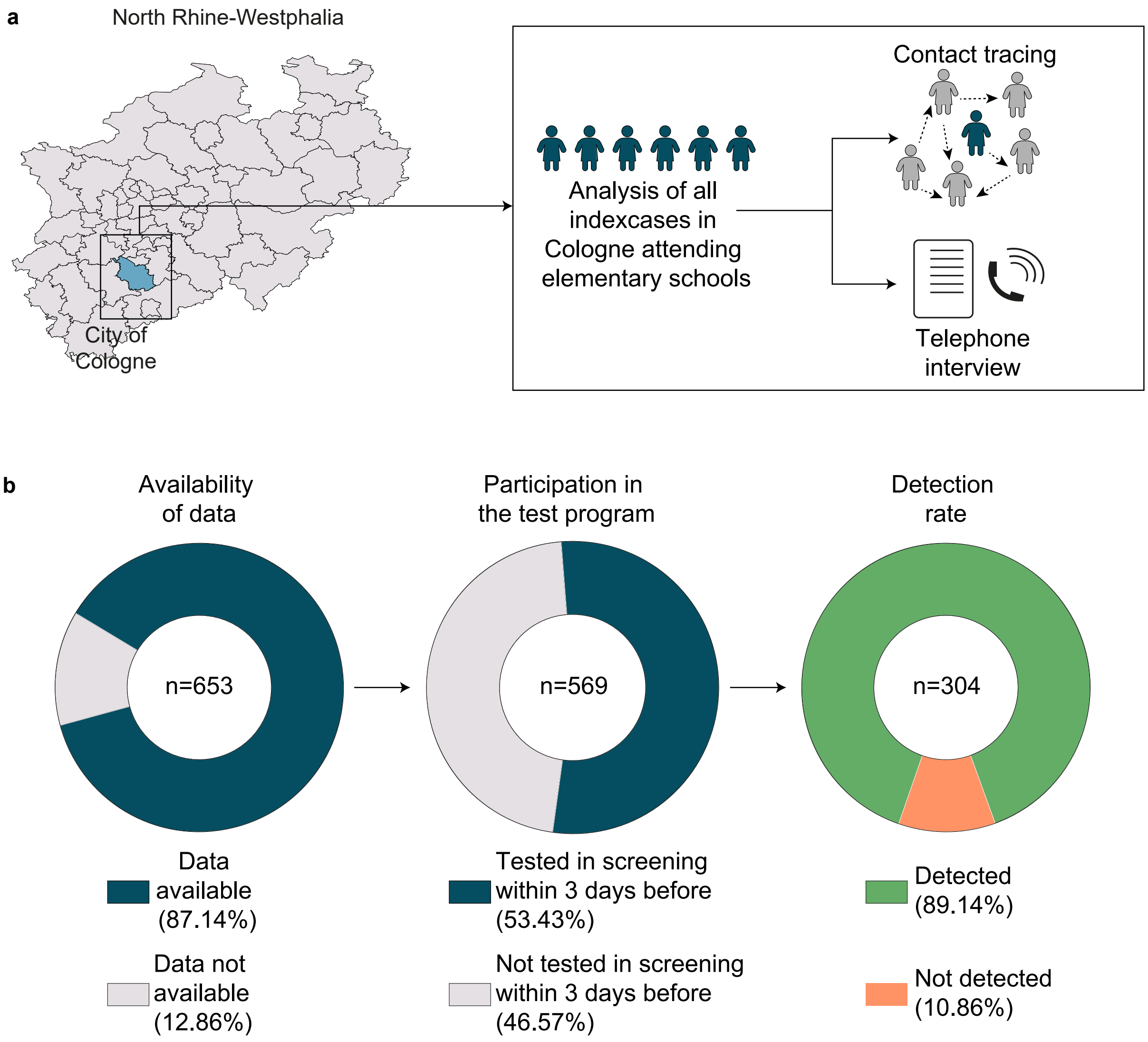

Supplementary Fig. 7: Determination of the SARS-CoV-2 detection rate of the Lolli-Method
a, Contact-tracing data of all index-cases attending elementary schools in the city of Cologne were analyzed.
b, Pie-chart categorizing all index-cases attending elementary schools in Cologne.

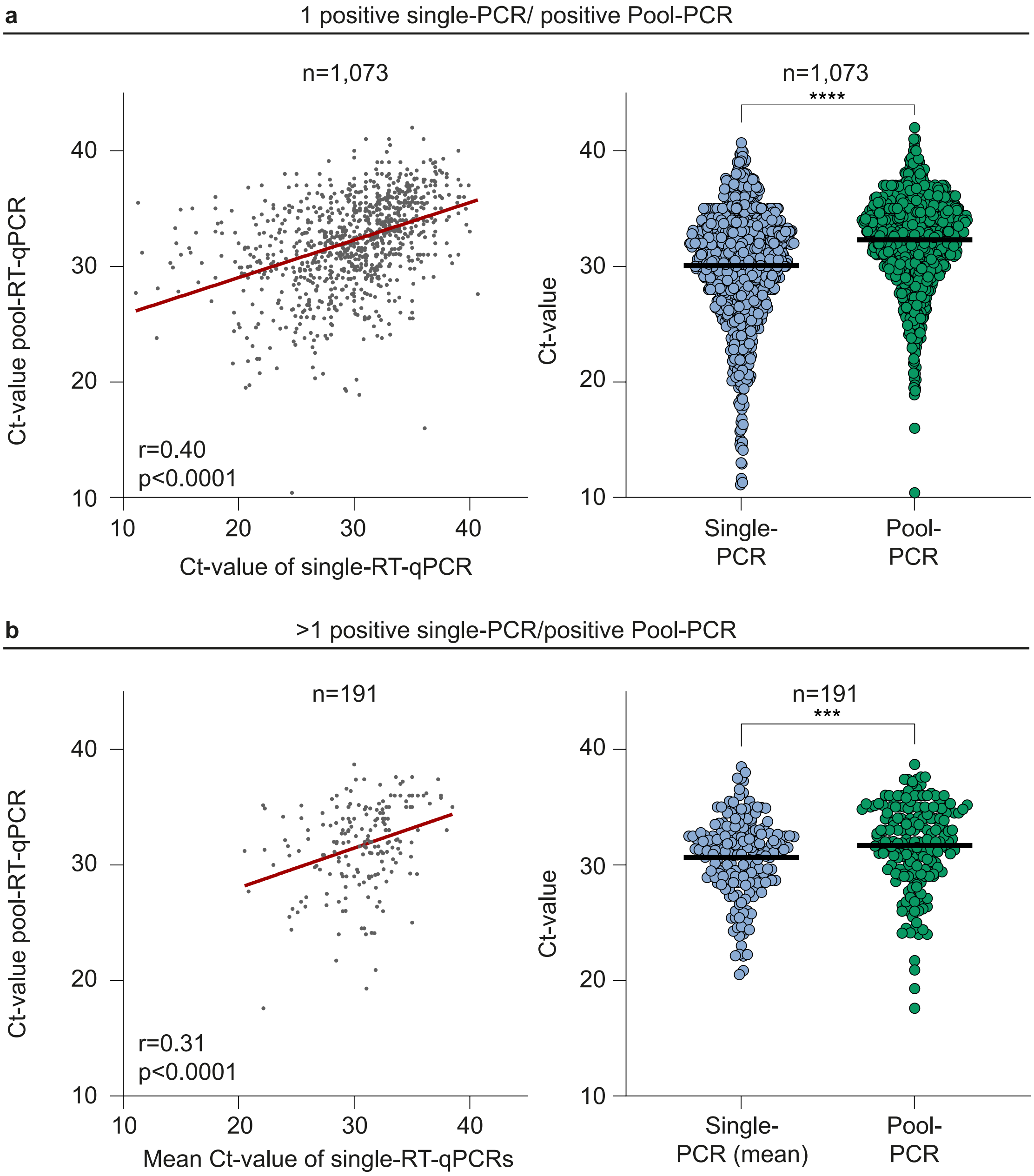

Supplementary Fig. 8: Correlation between Ct-values of pool-RT-qPCR and matched single-RT-qPCRs.
a, Left: Spearman correlation (two-tailed) of Ct-values of pool-RT-qPCRs containing only one positive single-RT-qPCR and the Ct-value of their matched single-RT-qPCR (n=1,073; r=0.4; p<0.0001). Right: Single- and pool-RT-qPCRs are plotted by Ct-value. Horizontal lines indicate mean Ct-values (p<0.0001, two-tailed WSR).**b**, Left: Spearman correlation (two-tailed) of Ct-values of pool-RT-qPCRs containing more than one positive single-RT-qPCR and the mean Ct-value of their matched single-RT-qPCRs (n=191; r=0.31, p<0,0001). Right: Single- and pool-RT-qPCRs are plotted by Ct-value/mean Ct-values. Horizontal lines indicate average Ct-values (p=0.0004, two-tailed WSR).

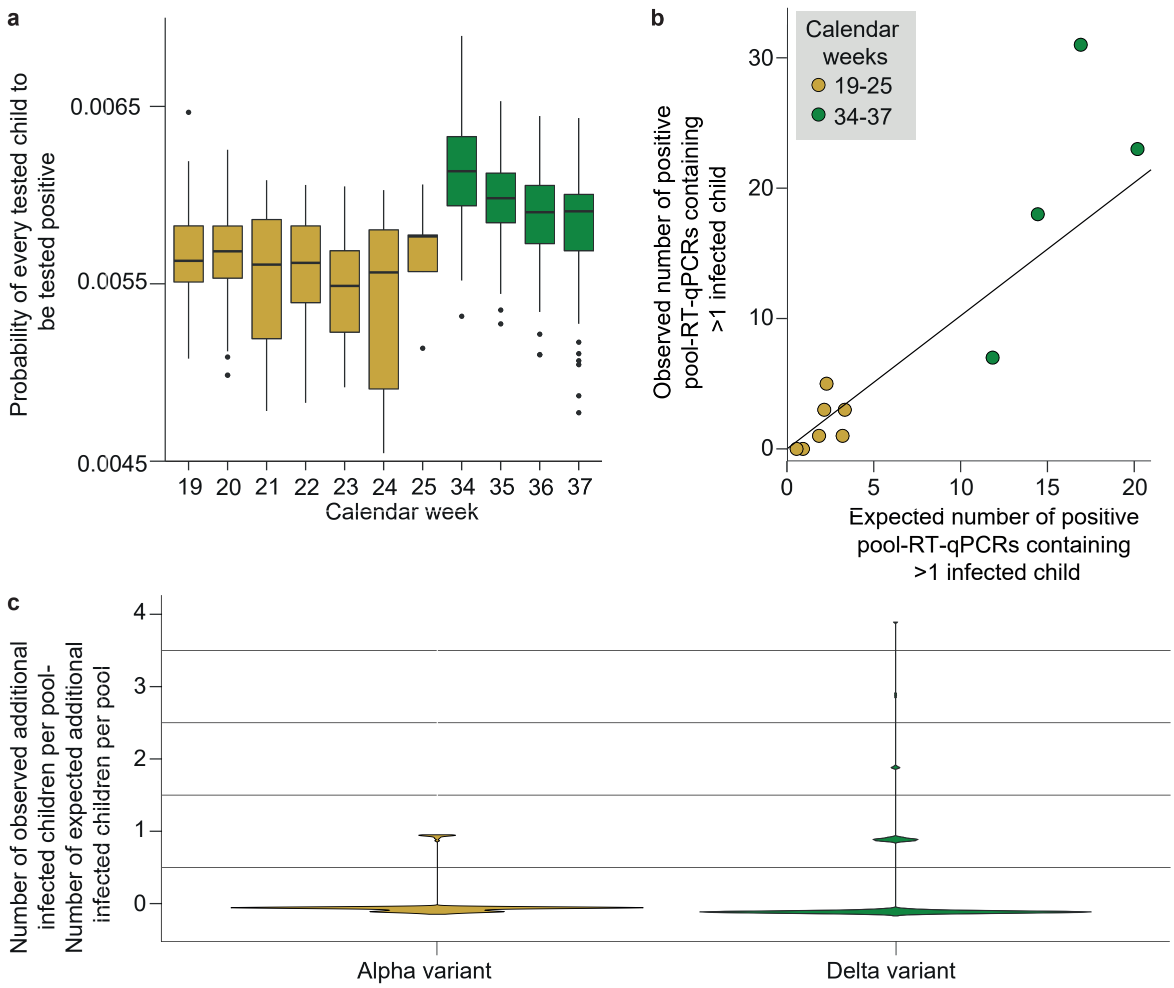

Supplementary Fig. 9: Modelling differences in infections dynamics of SARS-CoV-2 variants with the Lolli-Method in schools

a, Boxplot indicating the probability of every child to be tested positive stratified by calendar week. N=782 biologically independent positive pool-RT-qPCRs. Calendar weeks 19-25 (yellow boxes) und 34-37 (green boxes) are included in this plot. The horizontal lines indicate the medians, the lines at the top and at the bottom of the boxes indicate first and third quartiles, the vertical bars represent upper and lower whiskers and the dots represent outliers. **b**, Observed number stratified by expected number of positive pool-RT-qPCRs containing >1 infected child per calendar week. Calendar weeks 19-25 (yellow dots) and 34-37 (green dots) are included in this plot. **c**, Violin plot indicating the difference between observed and expected number of additional infected children per positive pool-RT-qPCR stratified by SARS-CoV-2 variant (p=0.002, WSR).

Supplementary Table 1

SEIR-model parameter description

Parameter	Description	Value	Reference
τ_{pre}	Time between infection and infectiousness	3 days	*40*
τ_{inf}	Duration of infectiousness	6 days	*41*
R0	Basic reproduction number	2,5	*42*
		4,5	*43,44*
		7,5	*37*
p_{in}		0,10%	*Assumption*
		0,01%	
p_{PCR}	False-negative rate of RT-qPCR	2,00%	*45,46,47*
g	Exponential growth rate of viral load	1/(4,5 days)	*48*

Supplementary Table 2

Tested schools and students

District	Schools (n)			Students (n)		
	Elementary schools	Schools for special needs	Other schools	Elementary schools	Schools for special needs	Other schools
**Aachen**	99	22	0	18629	3358	0
**Bielefeld**	51	13	0	12475	2292	0
**Bochum**	52	14	0	11584	3310	0
**Bonn**	55	8	1	12230	1352	416
**Borken**	74	11	0	14483	1598	0
**Bottrop**	20	3	0	4010	224	0
**Coesfeld**	40	5	0	8182	860	0
**Dortmund**	91	19	1	21496	2868	738
**Duisburg**	77	15	0	18417	2607	0
**Düren**	55	7	1	9272	1385	481
**Düsseldorf**	94	17	0	21777	3178	0
**Ennepe-Ruhr-Kreis**	56	8	1	10709	1340	375
**Essen**	87	22	0	20396	3865	0
**Euskirchen**	39	9	0	6729	1206	0
**Gelsenkirchen**	41	10	0	10411	1953	0
**Gütersloh**	69	15	0	13840	1809	0
**Hagen**	33	6	0	7186	1160	0
**Hamm**	28	6	1	6682	797	344
**Heinsberg**	55	7	0	9085	974	0
**Herford**	51	6	0	9230	1046	0
**Herne**	21	6	1	5593	656	1065
**Hochsauerlandkreis**	64	12	0	8756	1038	0
**Höxter**	25	6	0	4841	621	0
**Kleve**	58	11	0	11105	1513	0
**Krefeld**	34	8	1	7955	1459	487
**Köln**	159	28	0	38152	4844	0
**Leverkusen**	25	4	0	6457	399	0
**Lippe**	66	13	0	13229	1850	0
**Mettmann**	83	14	0	17856	1841	0
**Minden-Lübbecke**	64	14	2	11554	1477	998
**Märkischer Kreis**	70	11	1	14360	1822	518
**Mönchengladbach**	43	12	0	9408	1680	0
**Mülheim an der Ruhr**	24	4	0	6100	1067	0
**Münster**	47	8	2	9956	1798	516
**Oberbergischer Kreis**	51	11	0	10204	1783	0
**Oberhausen**	31	5	0	7202	715	0
**Olpe**	35	7	0	4885	1022	0
**Paderborn**	64	10	0	11768	1834	0
**Recklinghausen**	99	20	0	21906	3170	0
**Remscheid**	21	6	0	3976	509	0
**Rhein-Erft-Kreis**	78	15	0	17877	2135	0
**Rhein-Kreis Neuss**	79	9	0	17238	1400	0
**Rhein-Sieg-Kreis**	102	24	0	22715	3137	0
**Rheinisch-Bergischer Kreis**	56	8	0	10376	1165	0
**Siegen-Wittgenstein**	62	8	1	9868	875	333
**Soest**	58	13	1	10648	1862	280
**Solingen**	27	6	0	5608	622	0
**Steinfurt**	89	17	1	16816	2154	103
**Unna**	63	8	0	13860	1211	0
**Viersen**	48	8	1	10105	966	336
**Warendorf**	58	5	0	10431	768	0
**Wesel**	77	12	0	15722	2525	0
**Wuppertal**	57	11	2	13085	1558	688
**Total**	3105	577	18	646435	88658	7678

Supplementary Table 3

Equipment for SARS-CoV-2 screening in schools

Lab ID	RNA extraction	RT-qPCR equipment
**1**	1. Echolution Viral RNA/DNA Swab Kit (Bio Echo Life Science GmbH)	1. 2019-nCoV: RT-PCR Kit (BGI)<br>2. ABI 7500 / ABI 7500-fast / ABI 7300 (Applied Biosystems)
**2**	1. KingFisher Flex (ThermoFisher)<br>2. Tecan Freedom EVO-2 100 Base (Tecan)<br>3. Quick-DNA/RNA Viral MagBead (Zymo)	1. ViroQ Rapid SARS-CoV-2 (BAG Diagnostics)<br>2. CFX96 (BioRad)
**3**	Not available	1. LightCycler® 480 II (Roche Diagnostics)
**4**	1. Chemagen 360 (Perkin Elmer)	1. Cobas 480 Z II (Roche Diagnostics)<br>2. QuantStudio 5 (Thermofisher)<br>3. QuantStudio 7pro (Thermofisher)
**5**	1. Chemagen 360 (Perkin Elmer)	1. Hologic Panther Aptima Sars Co-V-2 Assay (Hologic)<br>2. Thermos TaqPath Covid-19 Kit (Thermofisher)<br>3. CFX 96 (BioRad)<br>4. CFX 384 (BioRad)
**6**	1. KingFisher (ThermoFisher)	1. Allplex™ SARS-CoV-2 Master Assay (Seegene)
**7**	1. KingFisher Flex (FA ThermoFisher)<br>2. Maelstrom 9600 (Tanbead)<br>3. RNA Extraction Kit AE1 (GSD NovaPrime®)	1. AriaDx (Agilent Technologies)<br>2. ABI 7500 FAST (FA ThermoFisher)<br>3. SARS-CoV-2 RT-PCR (GSD NovaPrime®)
**8**	1. Roche SARS-CoV-2 KIT (Roche Diagnostics)<br>2. AltoStar AM 16 (Altona Diganostics)	1. Cobas 6800 (Roche Diagnostics)<br>2. CFX 96 (BioRad)
**9**	Not available	Not available
**10**	1. Maelstrom 9600 (TanBead)	1. Allplex™ SARS-CoV-2 Master Assay (Seegene)
**11**	1. RoboPrep 96 (Biotecon Diagnostics)	1. CFX DX (BioRad)<br>2. LightCycler® 480 II (Roche Diagnostics)
**12**	1. Roche SARS-CoV-2 KIT (Roche Diagnostics)<br>2. KingFisher Flex (ThermoFisher)<br>3. ExtraStar 1.0, (Altona Diganostics)	1. Cobas 6800 (Roche Diagnostics)<br>2. Cobas 8800 (Roche Diagnostics)<br>3. CFX96 (BioRad)<br>4. RealStar SARS-CoV-2 RT-PCR Kit, (Altona Diganostics)

Description of additional Supplementary Files

File Name: Supplementary Data 1
Description: Validation of the Lolli Method (Morning and during the day)

File Name: Supplementary Data 2
Description: Validation of the Lolli Method (Before and 1 hour after breakfast)

File Name: Supplementary Data 3
Description: Validation of the Lolli Method (Different swab-types)

File Name: Supplementary Data 4
Description: Validation of the Lolli Method (Pooling)

File Name: Supplementary Data 5
Description: Validation of the Lolli Method (Specificity)

File Name: Supplementary Data 6
Description: Overview on SARS-CoV-2 screening in schools

File Name: Supplementary Data 7
Description: Code of the SEIR-model

File Name: Supplementary Data 8
Description: Code of the statistical modelling
