# Supplementary material for: Effective high-throughput RT-qPCR screening for SARS-CoV-2 infections in children
Source: Nat Commun. 2022 Jun 25;13:3640. doi: 10.1038/s41467-022-30664-2 (PMC9233713; doi:10.1038/s41467-022-30664-2)
Supplement: Supplementary file 3 — Description of Additional Supplementary Files [file 41467_2022_30664_MOESM3_ESM.pdf]

Description of additional Supplementary Files

File Name: Supplementary Data 1
Description: Validation of the Lolli Method (Morning and during the day)

File Name: Supplementary Data 2
Description: Validation of the Lolli Method (Before and 1 hour after breakfast)

File Name: Supplementary Data 3
Description: Validation of the Lolli Method (Different swab-types)

File Name: Supplementary Data 4
Description: Validation of the Lolli Method (Pooling)

File Name: Supplementary Data 5
Description: Validation of the Lolli Method (Specificity)

File Name: Supplementary Data 6
Description: Overview on SARS-CoV-2 screening in schools

File Name: Supplementary Data 7
Description: Code of the SEIR-model

File Name: Supplementary Data 8
Description: Code of the statistical modelling
