# Supplementary figures and images for: Effective high-throughput RT-qPCR screening for SARS-CoV-2 infections in children
Source: Nat Commun. 2022 Jun 25;13:3640. doi: 10.1038/s41467-022-30664-2 (PMC9233713; doi:10.1038/s41467-022-30664-2)

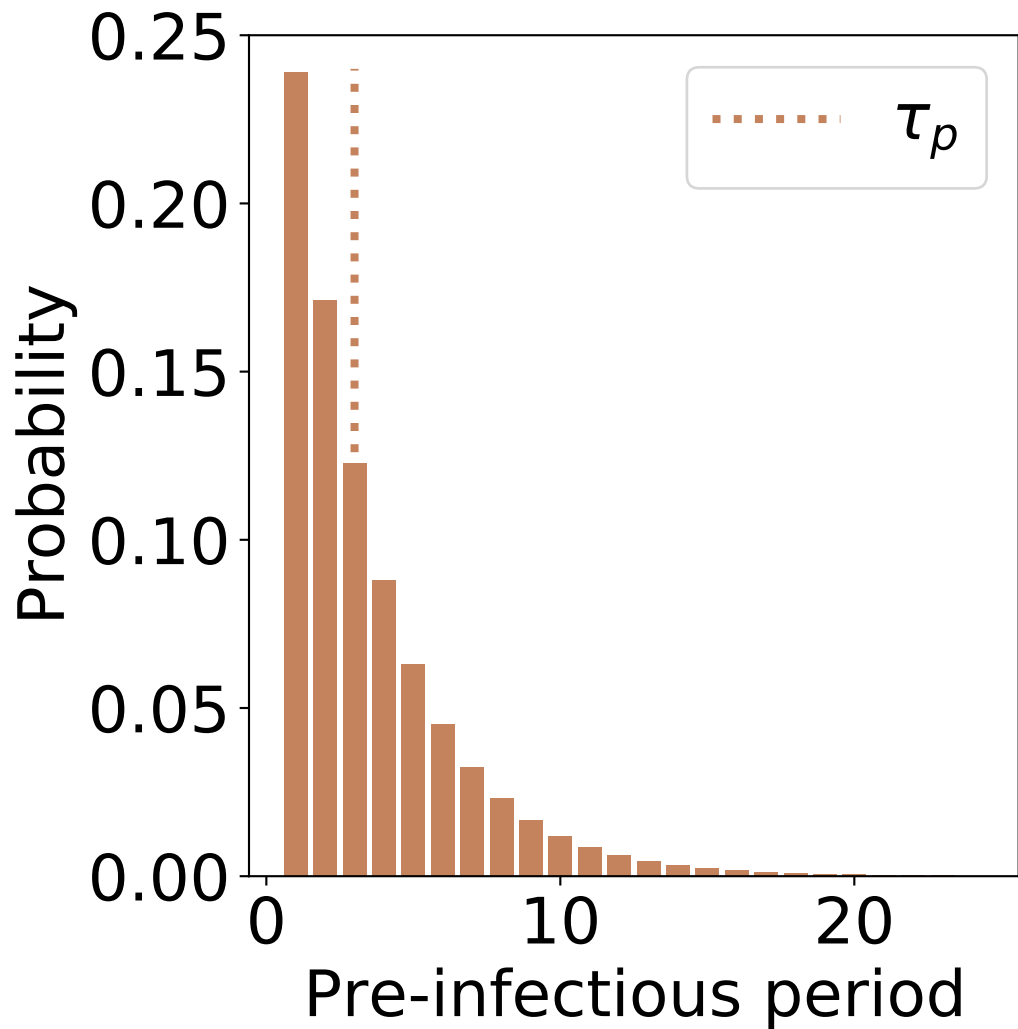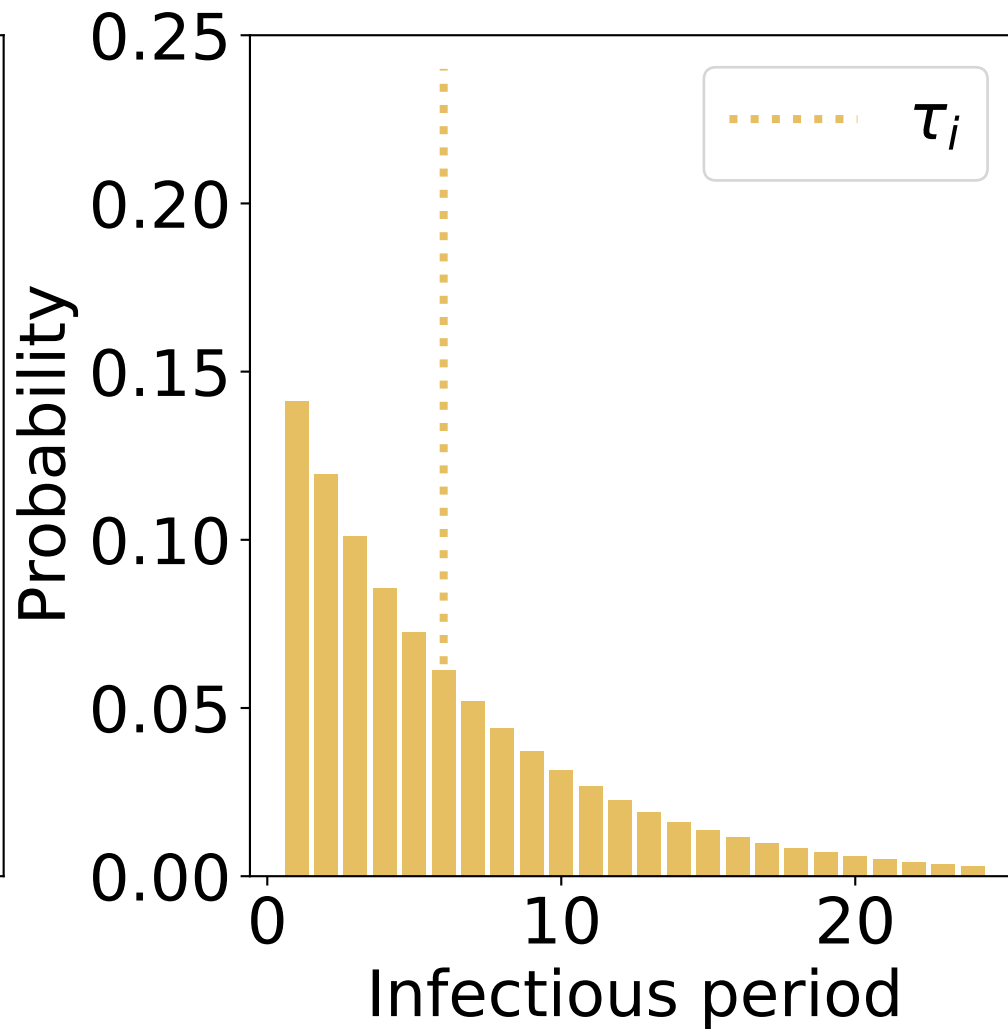

Supplement: Supplementary file 10 — Supplementary Data 7 [file 41467_2022_30664_MOESM10_ESM.zip › lolli_testing/Figures/tau_distributions.pdf]

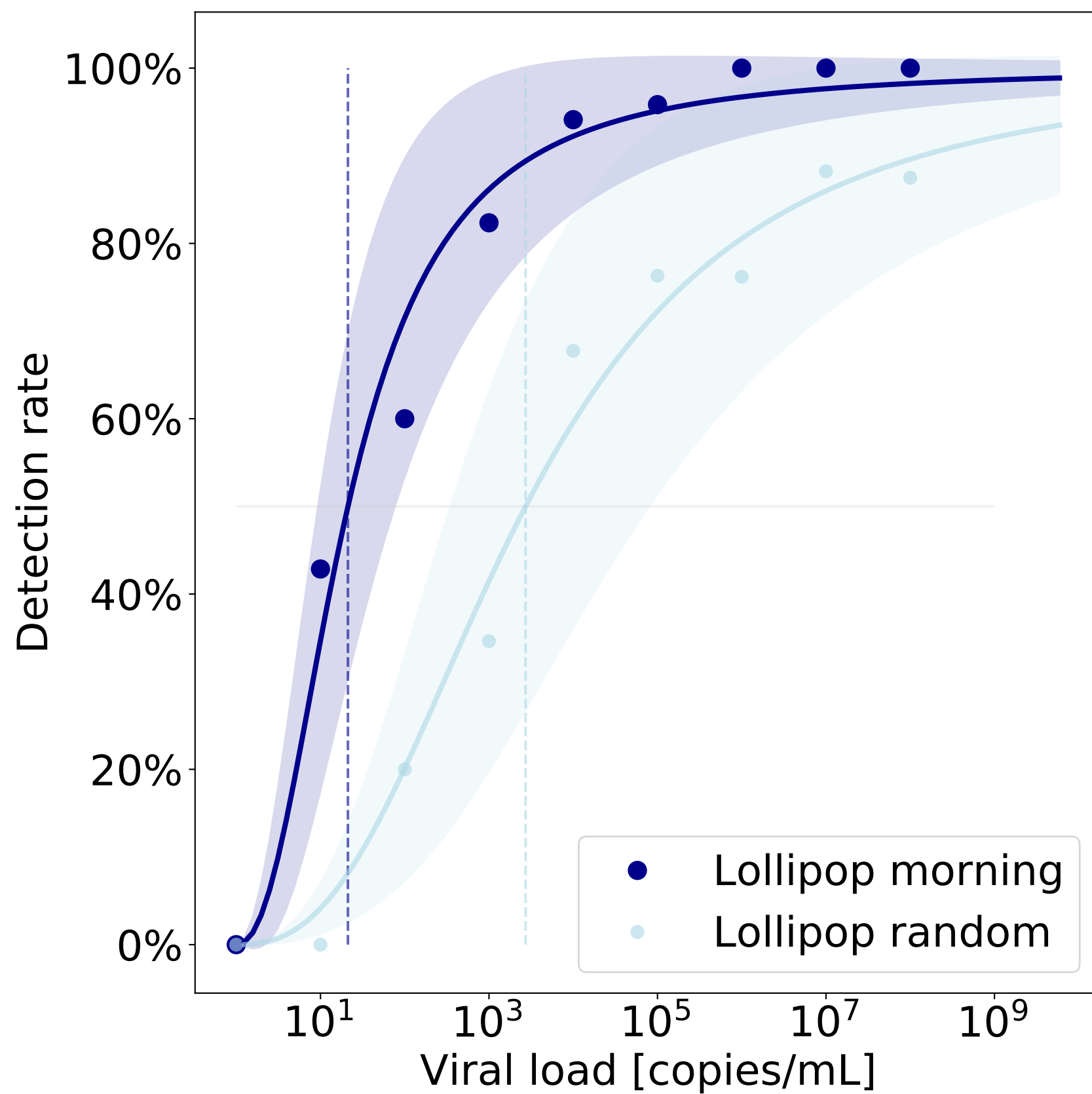

Supplement: Supplementary file 10 — Supplementary Data 7 [file 41467_2022_30664_MOESM10_ESM.zip › lolli_testing/Figures/0_Sensitivity/fit.pdf]

# Exponential growth

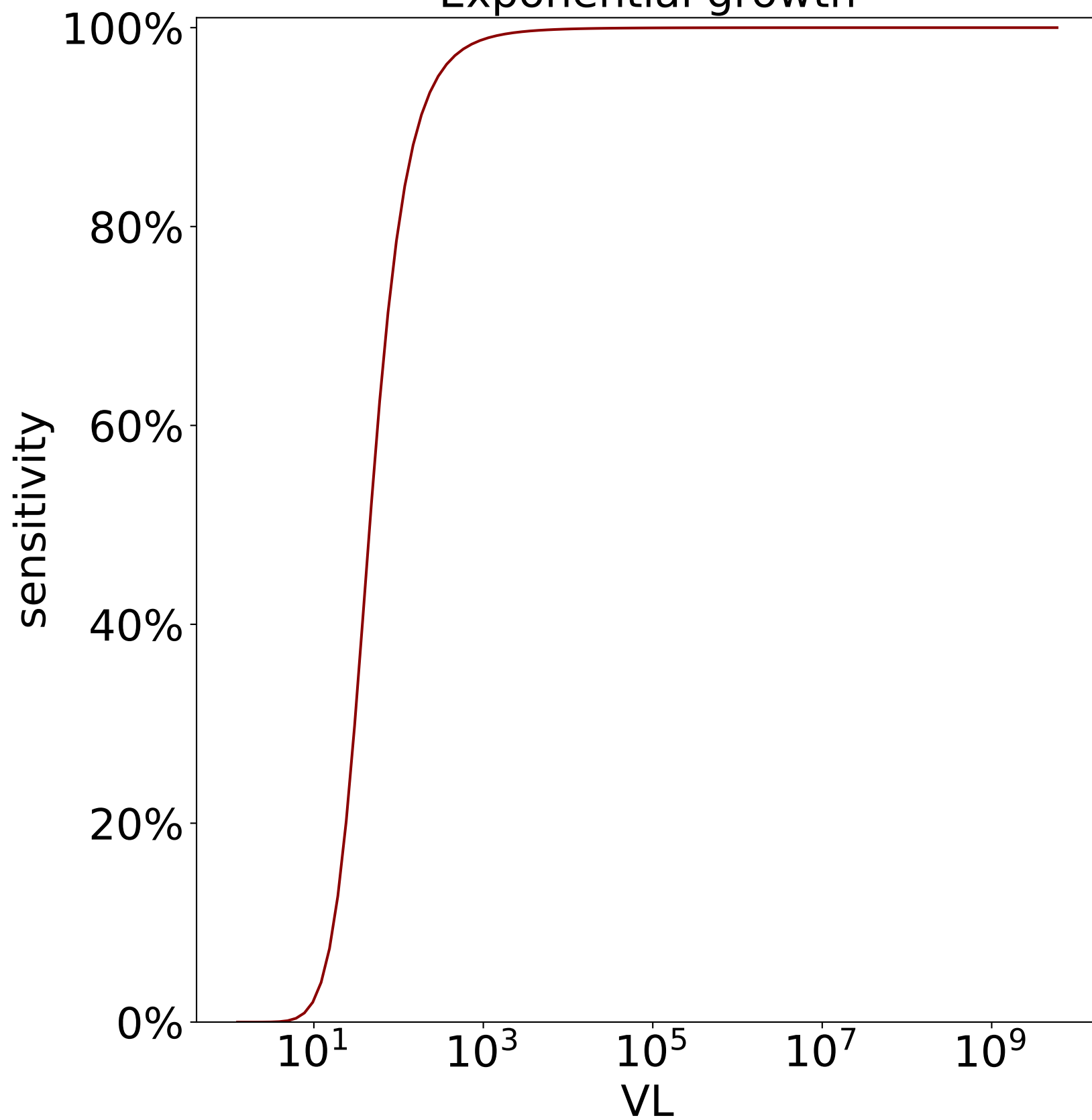

Supplement: Supplementary file 10 — Supplementary Data 7 [file 41467_2022_30664_MOESM10_ESM.zip › lolli_testing/Figures/0_Sensitivity/sensitivity_fit.pdf]

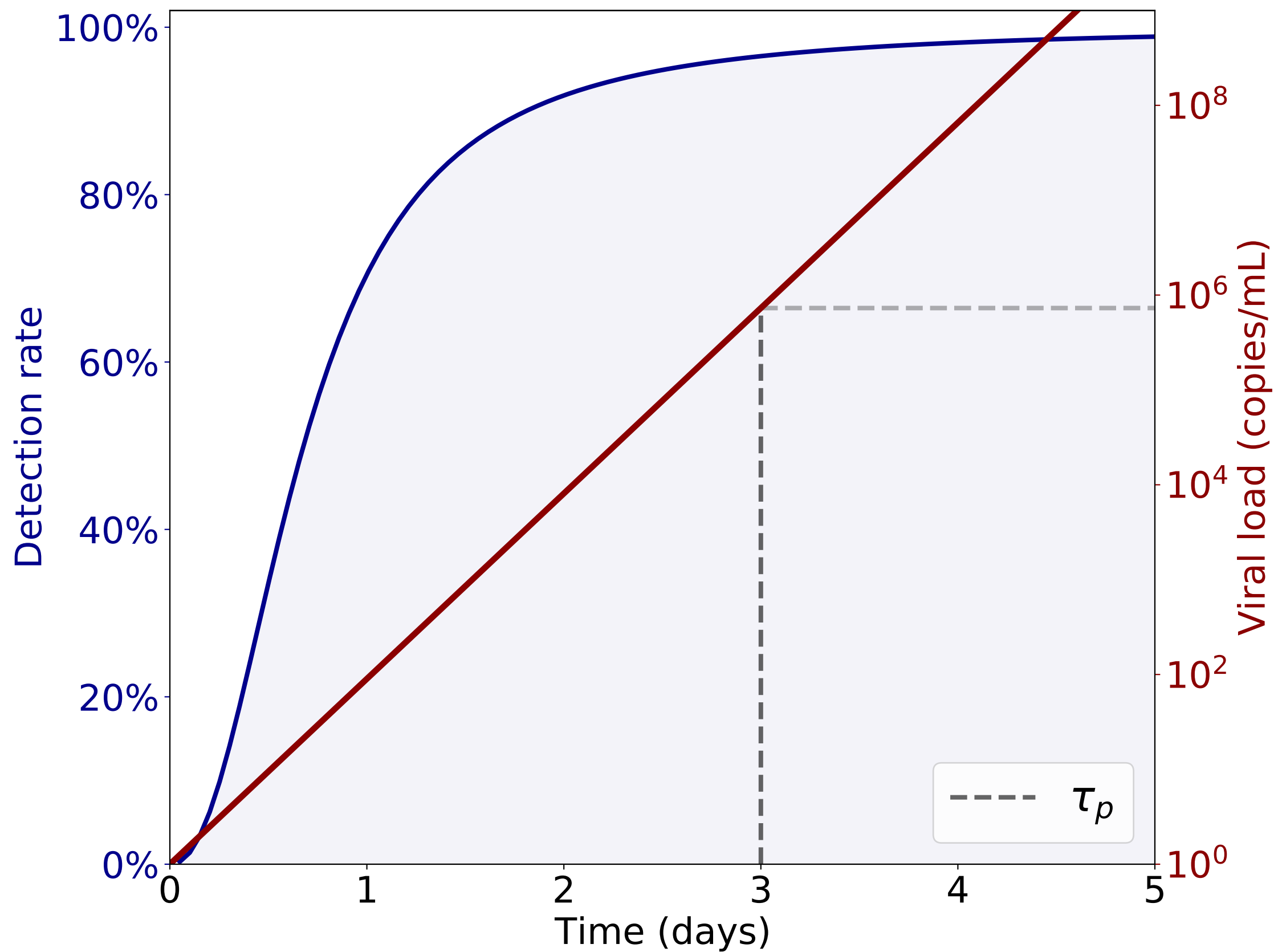

Supplement: Supplementary file 10 — Supplementary Data 7 [file 41467_2022_30664_MOESM10_ESM.zip › lolli_testing/Figures/0_Sensitivity/detection_rate_1.pdf]

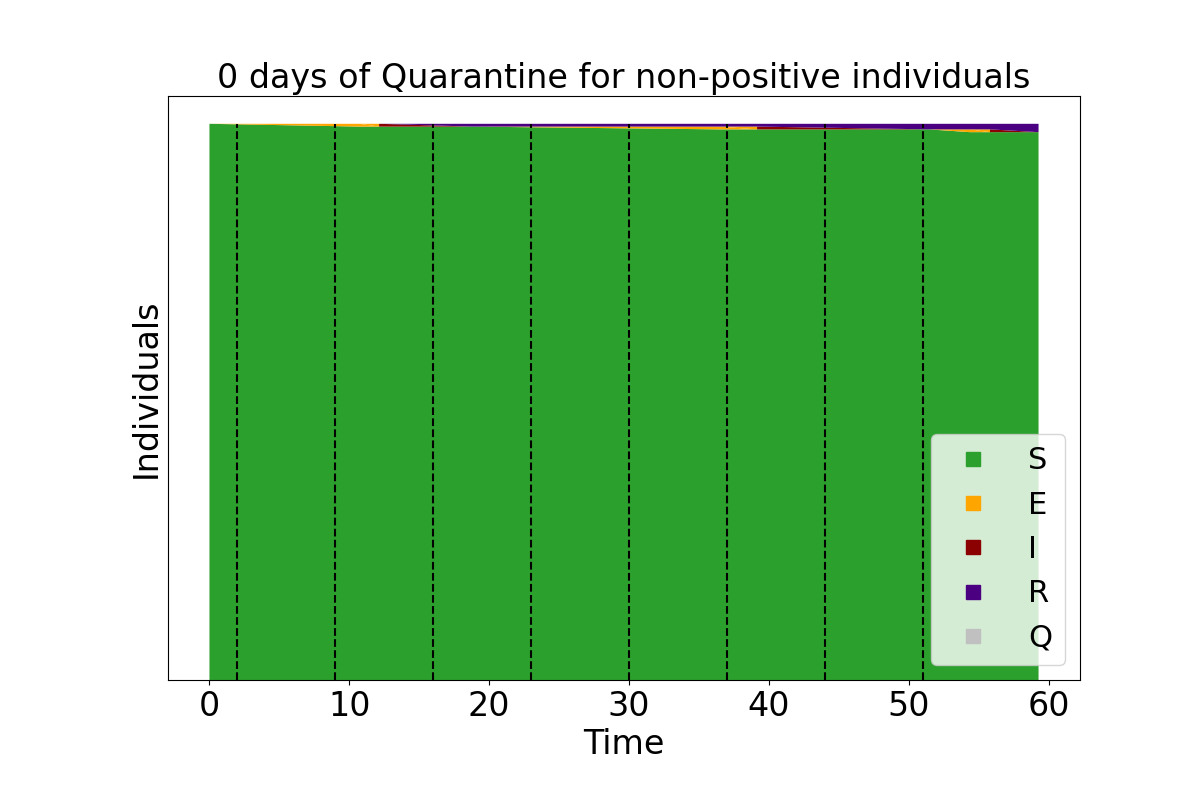

Supplement: Supplementary file 10 — Supplementary Data 7 [file 41467_2022_30664_MOESM10_ESM.zip › lolli_testing/Figures/1_Extended_Model/examples/population_R0-4.5_d-0_prev-1.0e-03.png]

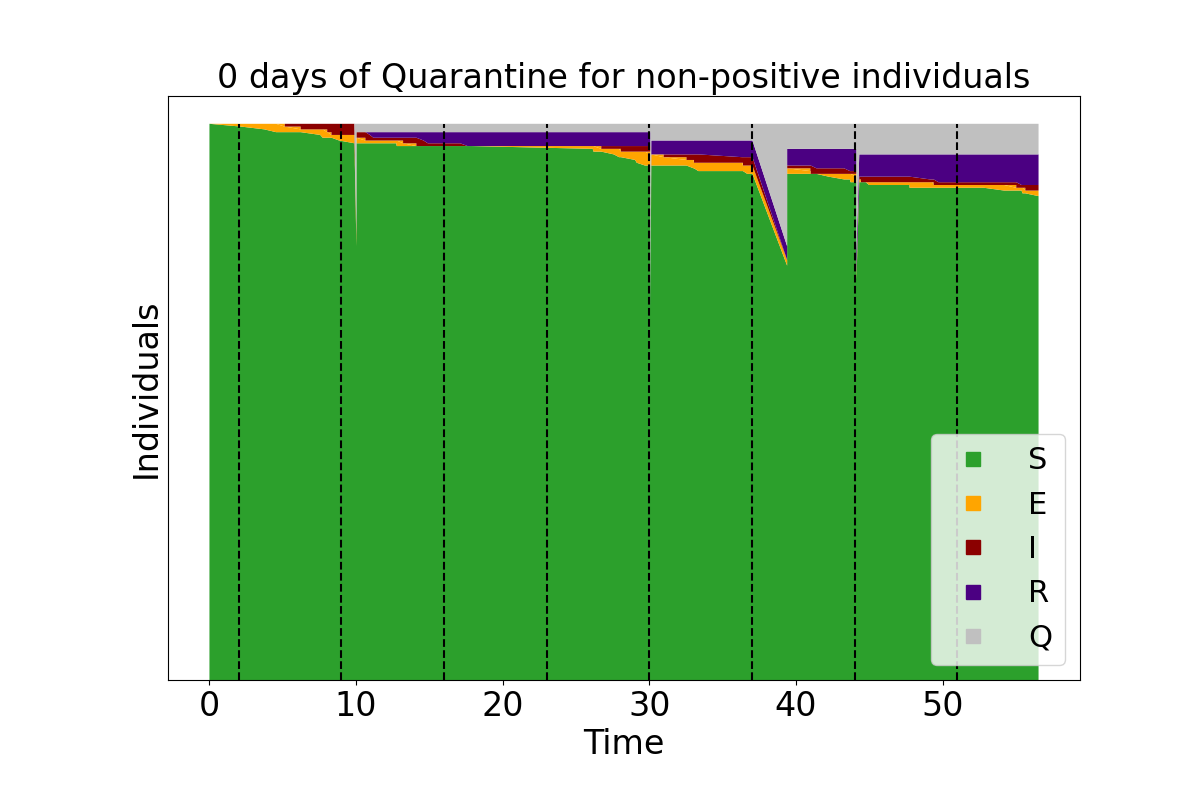

Supplement: Supplementary file 10 — Supplementary Data 7 [file 41467_2022_30664_MOESM10_ESM.zip › lolli_testing/Figures/1_Extended_Model/examples/population_R0-4.5_d-0_prev-1.0e-02.png]

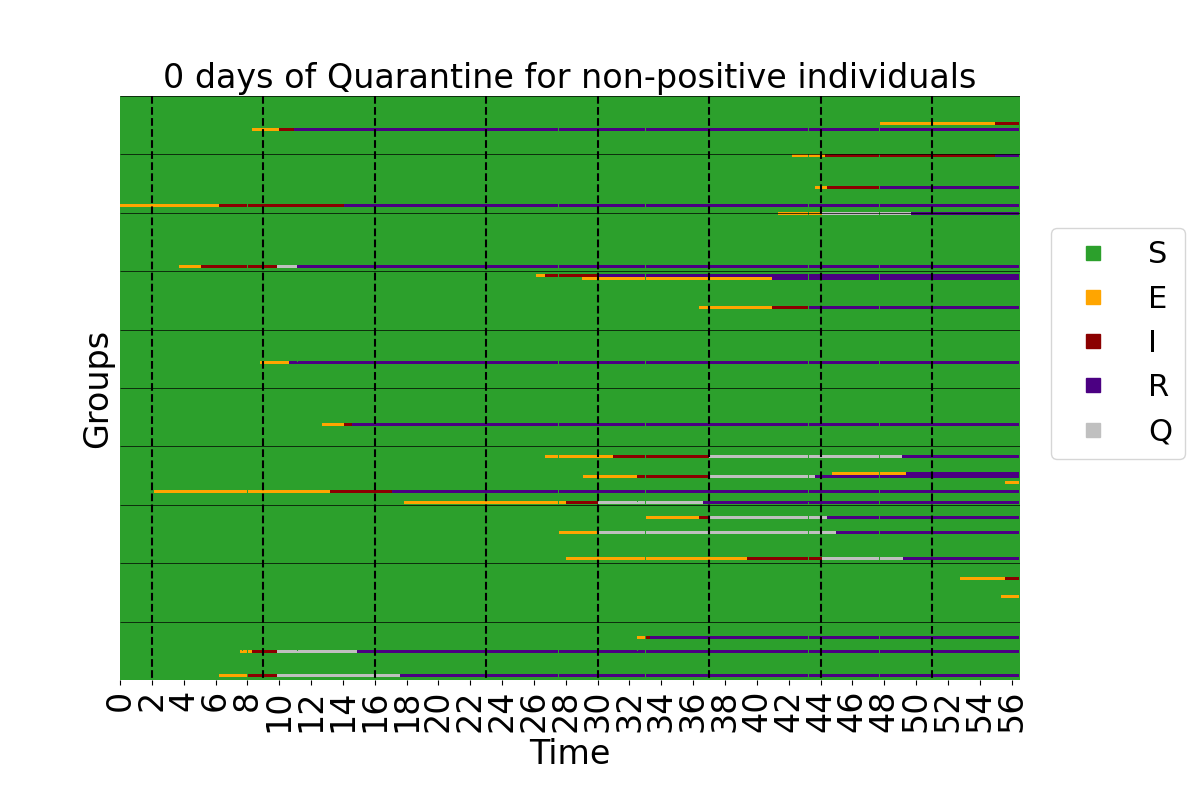

Supplement: Supplementary file 10 — Supplementary Data 7 [file 41467_2022_30664_MOESM10_ESM.zip › lolli_testing/Figures/1_Extended_Model/examples/groups_R0-4.5_d-0_prev-1.0e-02.png]

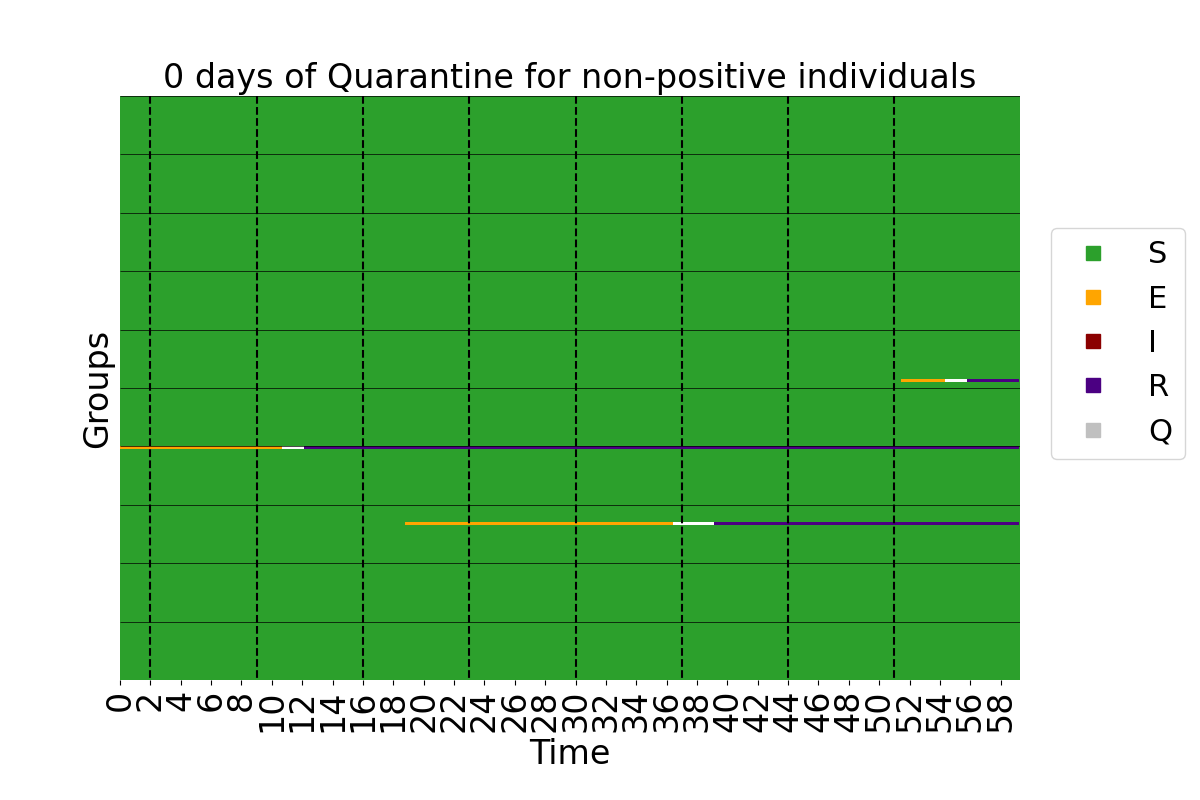

Supplement: Supplementary file 10 — Supplementary Data 7 [file 41467_2022_30664_MOESM10_ESM.zip › lolli_testing/Figures/1_Extended_Model/examples/groups_R0-4.5_d-0_prev-1.0e-03.png]

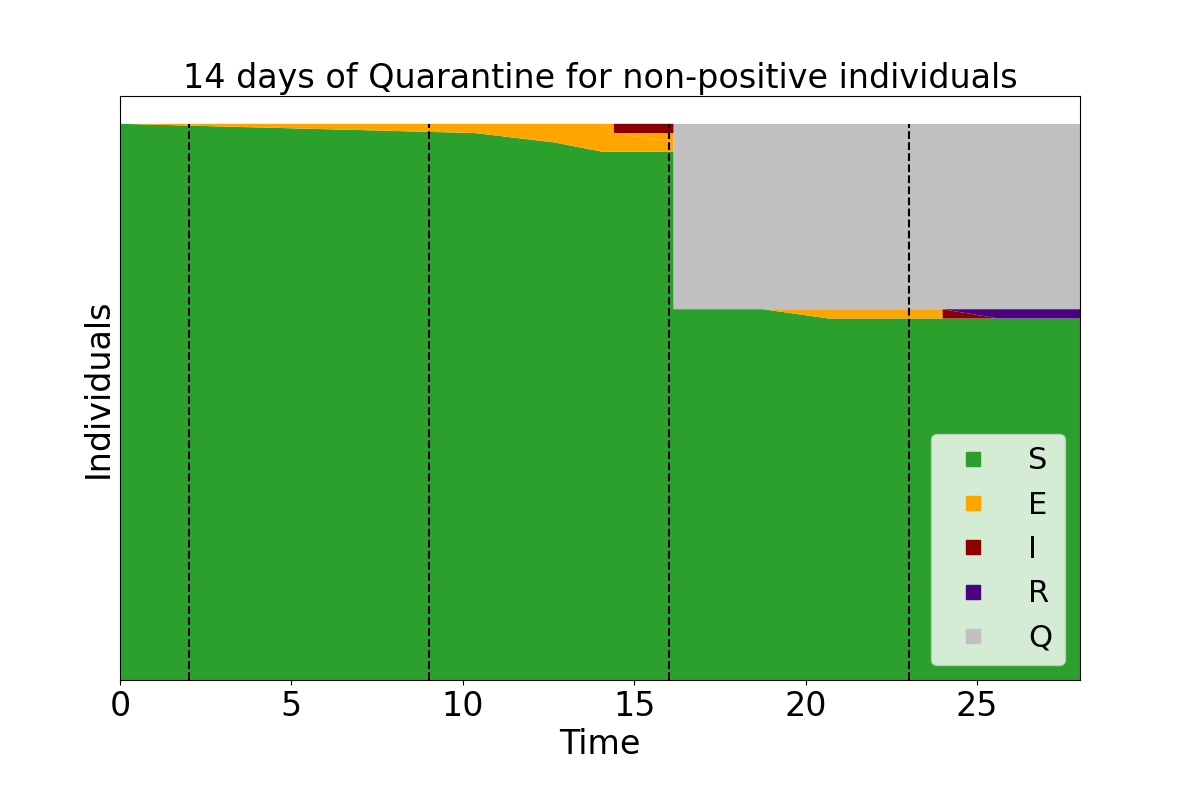

Supplement: Supplementary file 10 — Supplementary Data 7 [file 41467_2022_30664_MOESM10_ESM.zip › lolli_testing/Figures/1_Extended_Model/examples/population_R0-4.5_d-14_prev-1.0e-02_interactions-1.png]

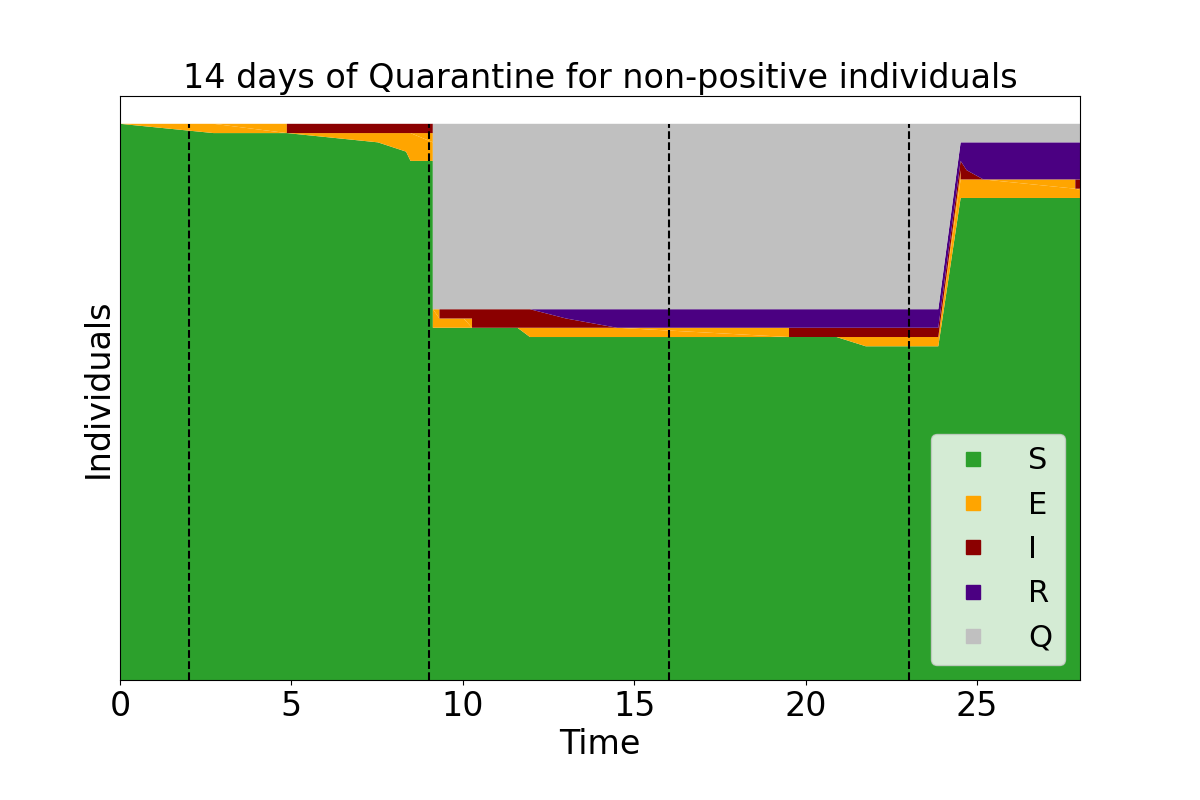

Supplement: Supplementary file 10 — Supplementary Data 7 [file 41467_2022_30664_MOESM10_ESM.zip › lolli_testing/Figures/1_Extended_Model/examples/population_R0-4.5_d-14_prev-1.0e-02_interactions-0.png]

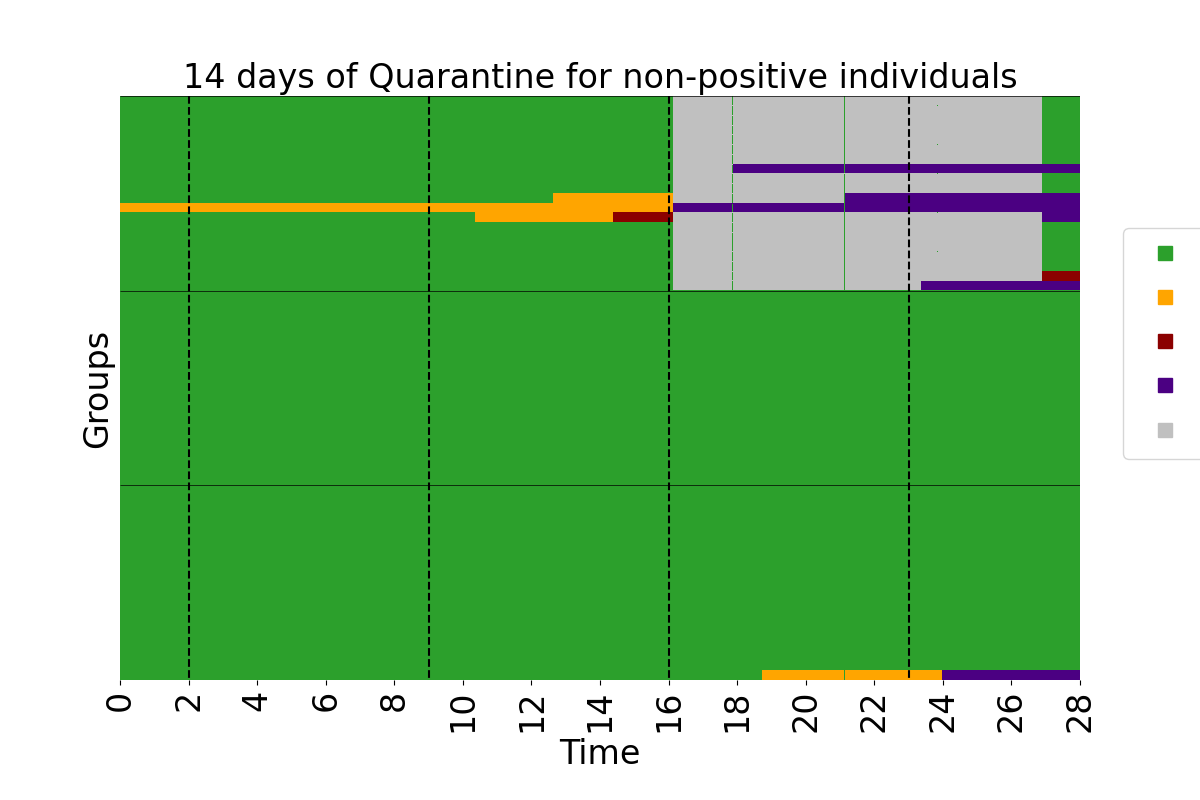

Supplement: Supplementary file 10 — Supplementary Data 7 [file 41467_2022_30664_MOESM10_ESM.zip › lolli_testing/Figures/1_Extended_Model/examples/groups_R0-4.5_d-14_prev-1.0e-02_interactions-1.png]

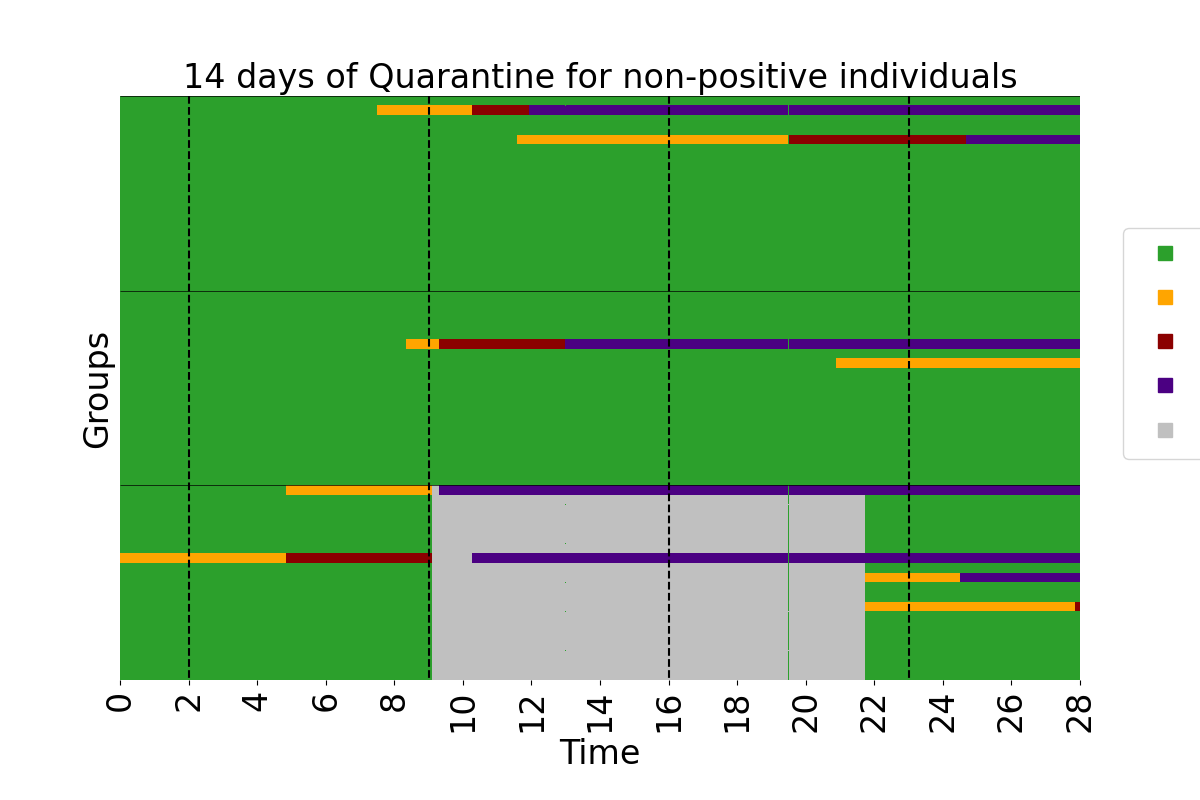

Supplement: Supplementary file 10 — Supplementary Data 7 [file 41467_2022_30664_MOESM10_ESM.zip › lolli_testing/Figures/1_Extended_Model/examples/groups_R0-4.5_d-14_prev-1.0e-02_interactions-0.png]

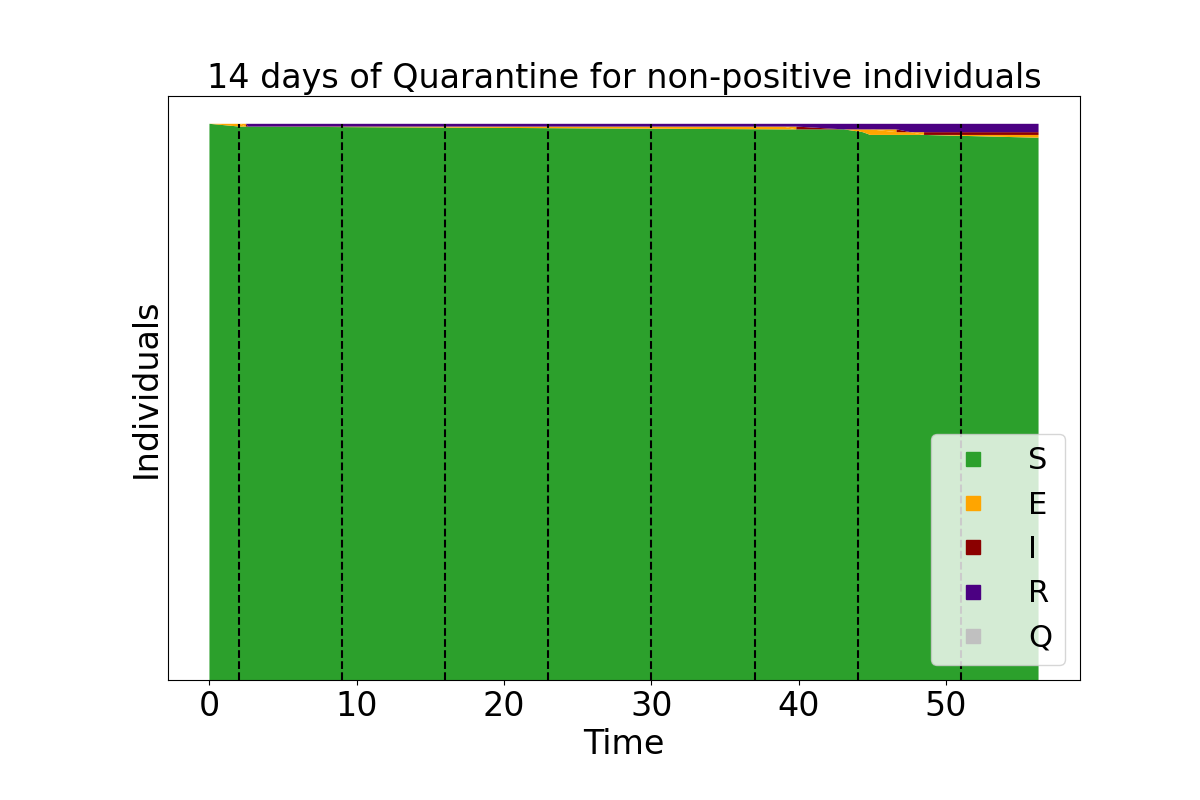

Supplement: Supplementary file 10 — Supplementary Data 7 [file 41467_2022_30664_MOESM10_ESM.zip › lolli_testing/Figures/1_Extended_Model/examples/population_R0-4.5_d-14_prev-1.0e-03_interactions-0.png]

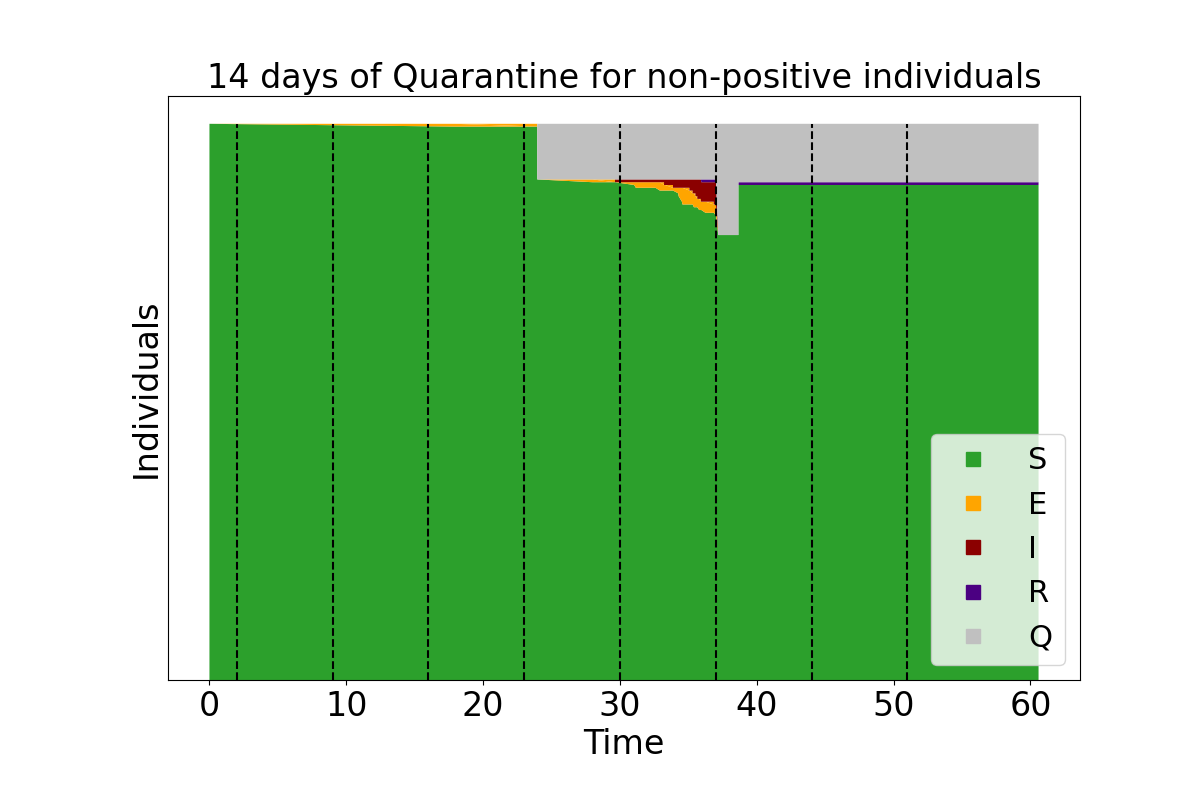

Supplement: Supplementary file 10 — Supplementary Data 7 [file 41467_2022_30664_MOESM10_ESM.zip › lolli_testing/Figures/1_Extended_Model/examples/population_R0-4.5_d-14_prev-1.0e-03_interactions-1.png]

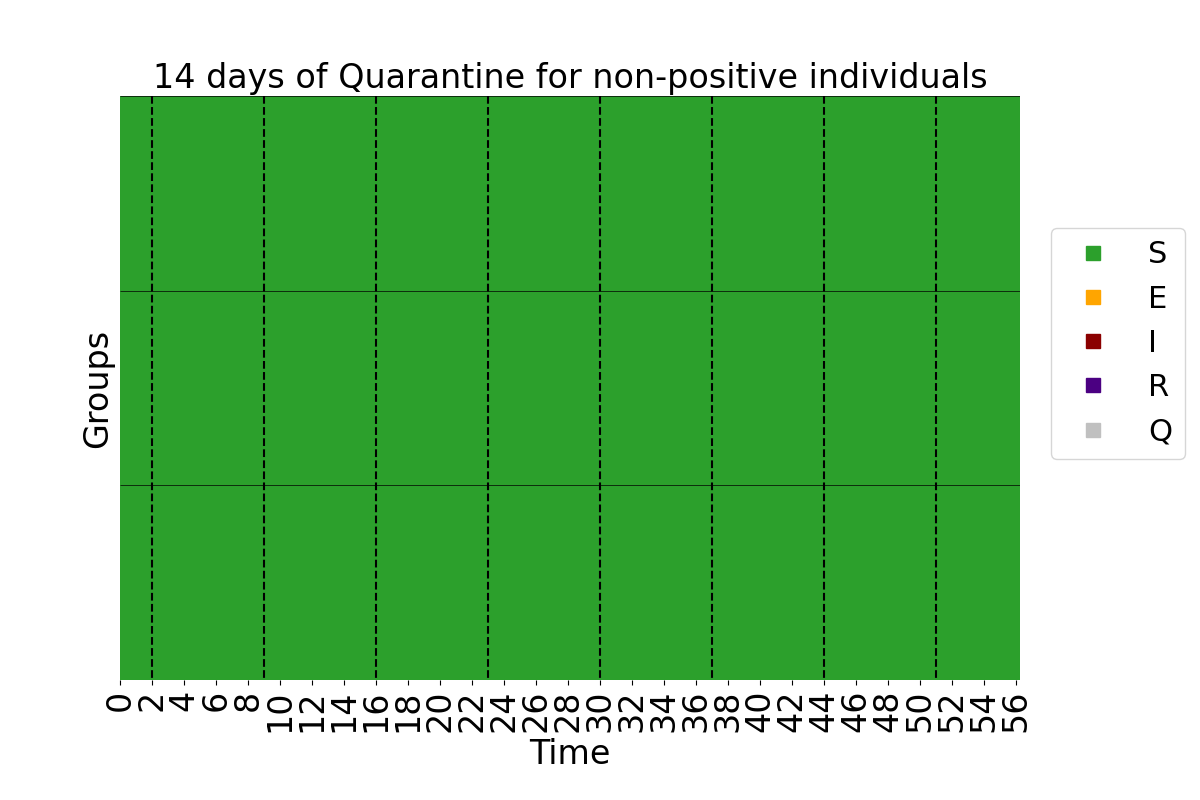

Supplement: Supplementary file 10 — Supplementary Data 7 [file 41467_2022_30664_MOESM10_ESM.zip › lolli_testing/Figures/1_Extended_Model/examples/groups_R0-4.5_d-14_prev-1.0e-03.png]

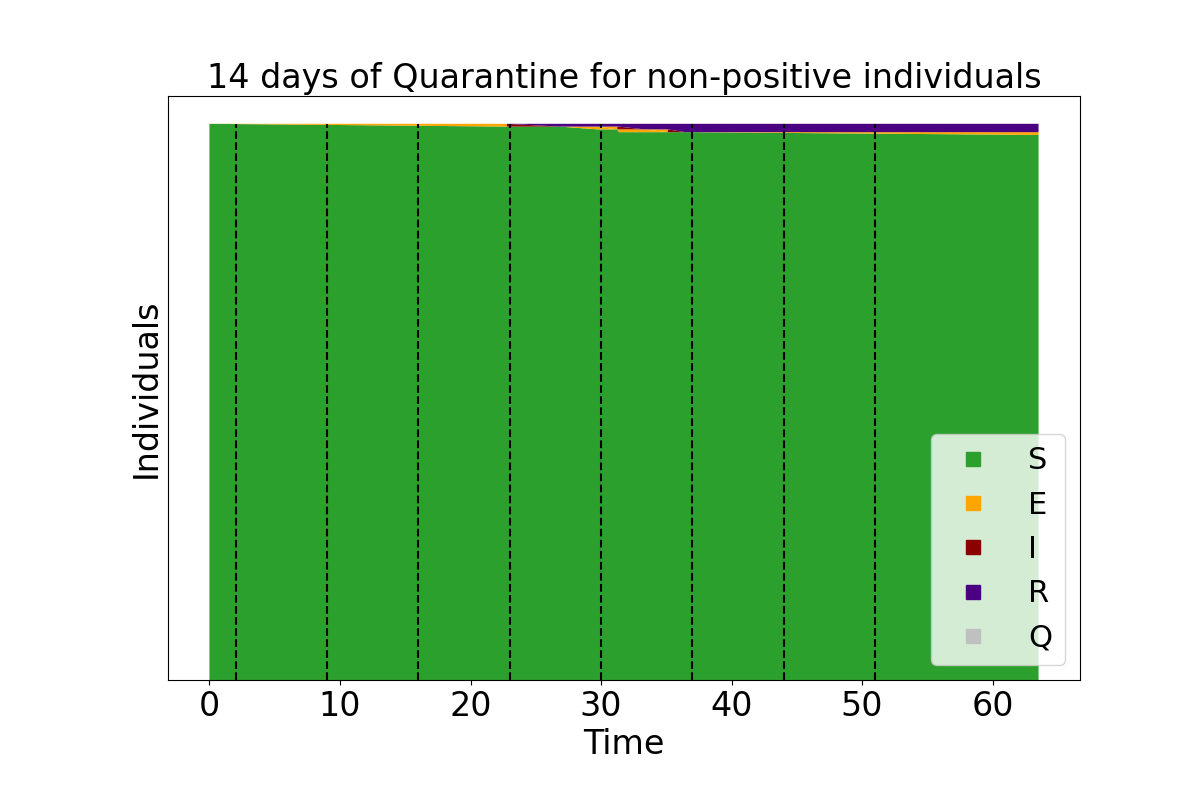

Supplement: Supplementary file 10 — Supplementary Data 7 [file 41467_2022_30664_MOESM10_ESM.zip › lolli_testing/Figures/1_Extended_Model/examples/population_R0-4.5_d-14_prev-1.0e-03.png]

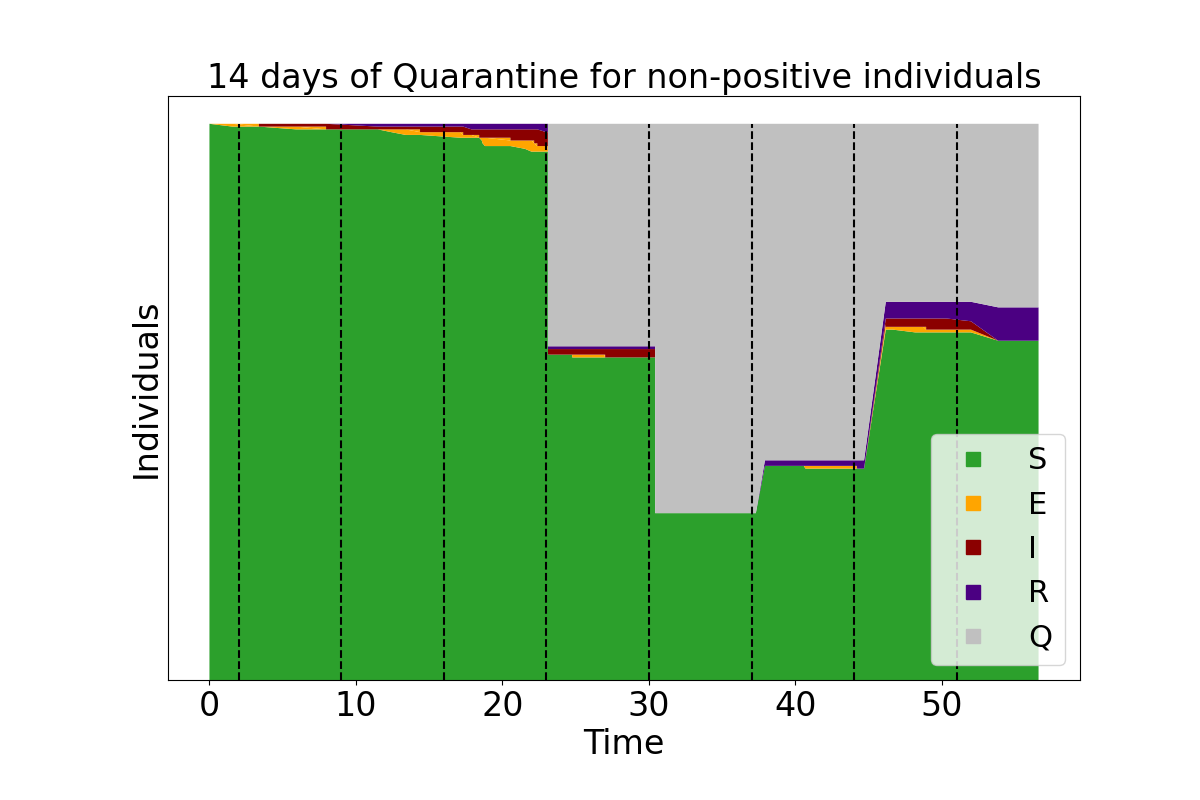

Supplement: Supplementary file 10 — Supplementary Data 7 [file 41467_2022_30664_MOESM10_ESM.zip › lolli_testing/Figures/1_Extended_Model/examples/population_R0-4.5_d-14_prev-1.0e-02.png]

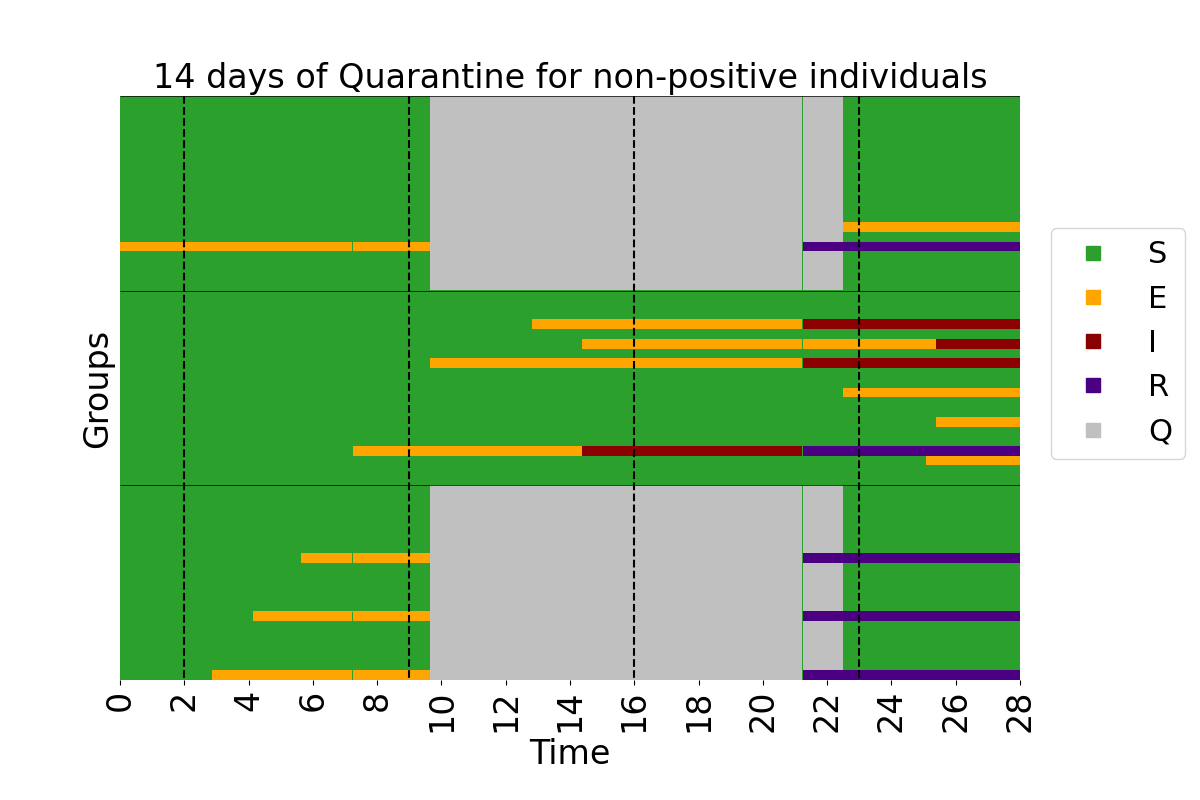

Supplement: Supplementary file 10 — Supplementary Data 7 [file 41467_2022_30664_MOESM10_ESM.zip › lolli_testing/Figures/1_Extended_Model/examples/example.png]

# 14 Isolations days

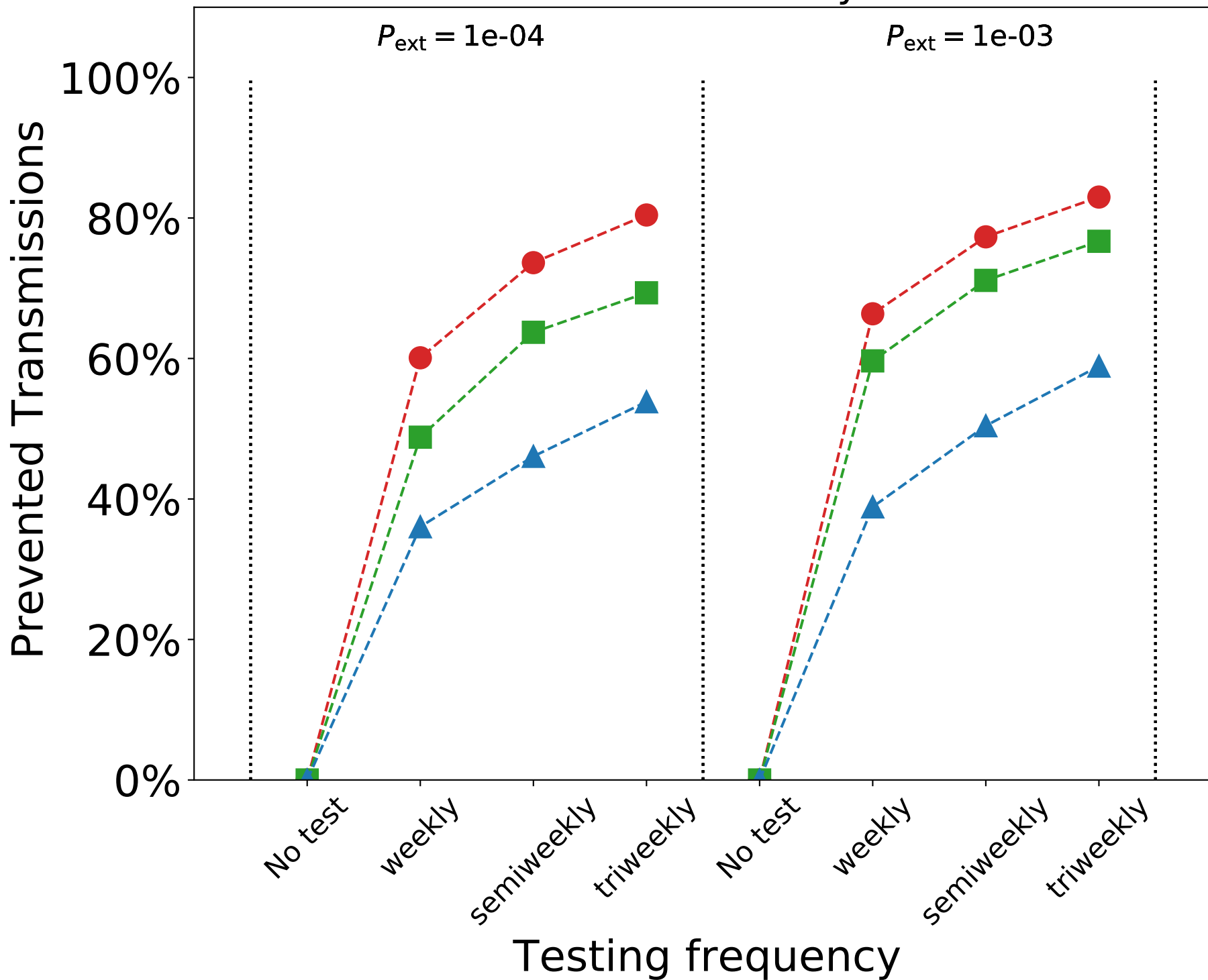

Supplement: Supplementary file 10 — Supplementary Data 7 [file 41467_2022_30664_MOESM10_ESM.zip › lolli_testing/Figures/1_Extended_Model/ensemble/1/PrevTrans_d-14.pdf]

# 0 Isolations days

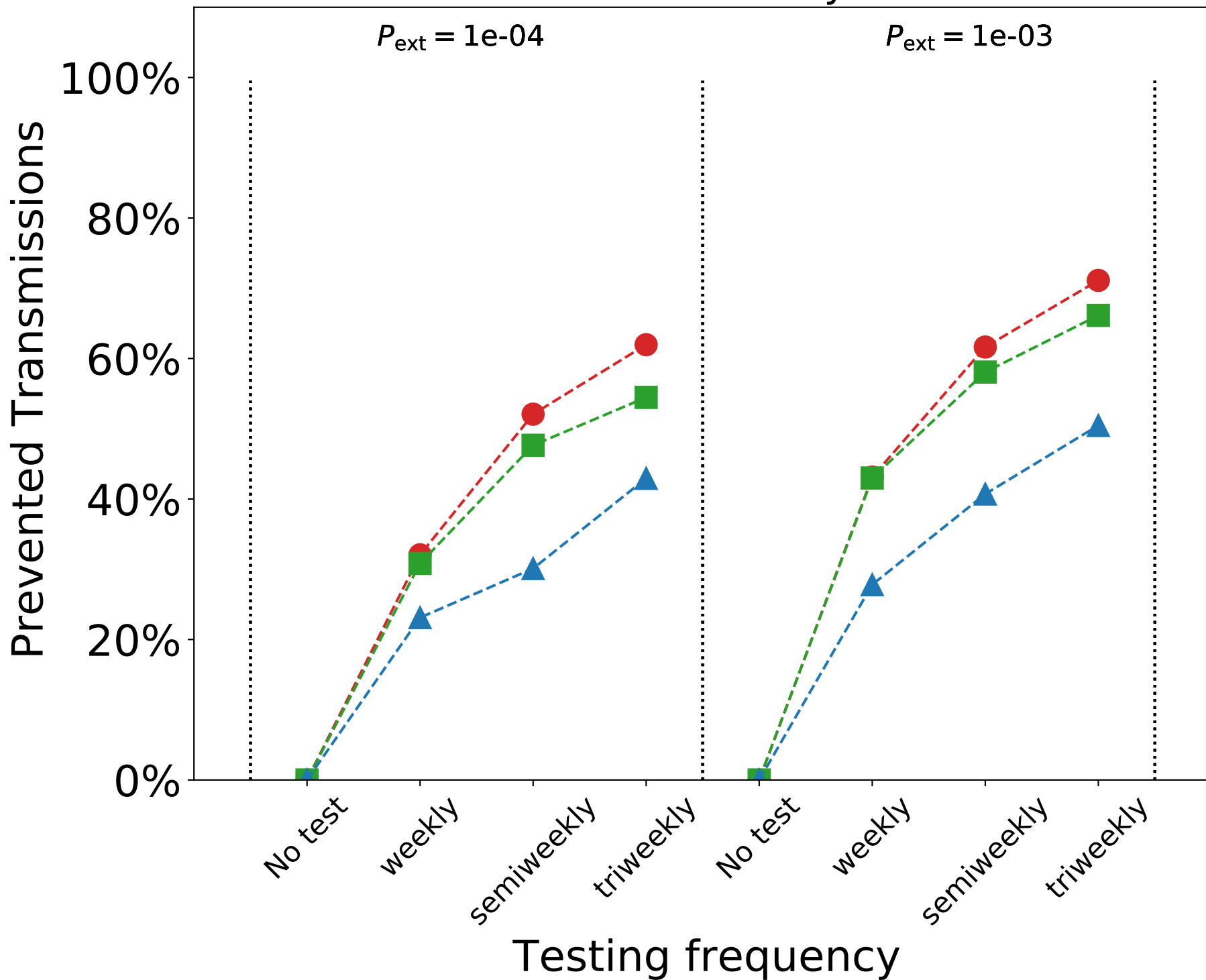

Supplement: Supplementary file 10 — Supplementary Data 7 [file 41467_2022_30664_MOESM10_ESM.zip › lolli_testing/Figures/1_Extended_Model/ensemble/1/PrevTrans_d-0.pdf]
