# Supplementary material for: Effective high-throughput RT-qPCR screening for SARS-CoV-2 infections in children
Source: Nat Commun. 2022 Jun 25;13:3640. doi: 10.1038/s41467-022-30664-2 (PMC9233713; doi:10.1038/s41467-022-30664-2)
Supplement: Supplementary file 12 — Reporting Summary [file 41467_2022_30664_MOESM12_ESM.pdf]

nature portfolio

Corresponding author(s): Florian Klein

Last updated by author(s): Apr 26, 2022

Reporting Summary

Nature Portfolio wishes to improve the reproducibility of the work that we publish. This form provides structure for consistency and transparency in reporting. For further information on Nature Portfolio policies, see our Editorial Policies and the Editorial Policy Checklist.

Statistics

For all statistical analyses, confirm that the following items are present in the figure legend, table legend, main text, or Methods section.

n/a	Confirmed	
☐	☒	The exact sample size (*n*) for each experimental group/condition, given as a discrete number and unit of measurement
☐	☒	A statement on whether measurements were taken from distinct samples or whether the same sample was measured repeatedly
☐	☒	The statistical test(s) used AND whether they are one- or two-sided<br>*Only common tests should be described solely by name; describe more complex techniques in the Methods section.*
☐	☒	A description of all covariates tested
☒	☐	A description of any assumptions or corrections, such as tests of normality and adjustment for multiple comparisons
☐	☒	A full description of the statistical parameters including central tendency (e.g. means) or other basic estimates (e.g. regression coefficient) AND variation (e.g. standard deviation) or associated estimates of uncertainty (e.g. confidence intervals)
☐	☒	For null hypothesis testing, the test statistic (e.g. *F*, *t*, *r*) with confidence intervals, effect sizes, degrees of freedom and *P* value noted<br>*Give P values as exact values whenever suitable.*
☒	☐	For Bayesian analysis, information on the choice of priors and Markov chain Monte Carlo settings
☒	☐	For hierarchical and complex designs, identification of the appropriate level for tests and full reporting of outcomes
☐	☒	Estimates of effect sizes (e.g. Cohen's *d*, Pearson's *r*), indicating how they were calculated

Our web collection on statistics for biologists contains articles on many of the points above.

Software and code

Policy information about availability of computer code

Data collection	Data collection was done using the software R programing language (v. 3.5.2, stats package).
Data analysis	Data analysis was done using the software GraphPad Prism (v.9), Microsoft Excel for Mac (v.14.7.3.) and R programing language (v. 3.5.2, stats package).

For manuscripts utilizing custom algorithms or software that are central to the research but not yet described in published literature, software must be made available to editors and reviewers. We strongly encourage code deposition in a community repository (e.g. GitHub). See the Nature Portfolio guidelines for submitting code & software for further information.

Data

Policy information about availability of data

All manuscripts must include a data availability statement. This statement should provide the following information, where applicable:

- Accession codes, unique identifiers, or web links for publicly available datasets
- A description of any restrictions on data availability
- For clinical datasets or third party data, please ensure that the statement adheres to our policy

The data that support the findings of this study are provided with this paper. Source data are provided with this paper.

Field-specific reporting

Please select the one below that is the best fit for your research. If you are not sure, read the appropriate sections before making your selection.

☒ Life sciences ☐ Behavioural & social sciences ☐ Ecological, evolutionary & environmental sciences

For a reference copy of the document with all sections, see nature.com/documents/nr-reporting-summary-flat.pdf

Life sciences study design

All studies must disclose on these points even when the disclosure is negative.

Sample size

Prospective validation study:
A priori power analysis was performed. Based on that, a paired t-test requires 28 participants to detect a standardized difference of 0.5 with a correlation of 0.7 (of the two measurements) and two-tailed type I error (alpha) of 5% with a power of 90% (1- beta). Due to feasibility and availability of acutely infected individuals, we decided to further confirm results by including in total 254 participants to strengthen the analysis.

Retrospective analysis of the SARS-CoV-2 screening in schools:
Because of the nature of this retrospective observational analysis, no power analysis could be performed. Testing was carried out at every elementary school and school for special needs all over North Rhine-Westphalia. Since participating in the screening program was mandatory for all students, we expect that all students (>730,000 students) were tested on a regular basis and therefore we think the number of tested individuals is sufficient.

Retrospective analysis of the SARS-CoV-2 screening in daycare facilities:
Because of the nature of this retrospective observational analysis, no power analysis could be performed. 698 of 700 daycare facilities in Cologne participated in the screening program. Eventhough testing was not mandatory, based on intensive communication with the daycare facilities and the Youth Welfare Office of the city of Cologne, we estimate a participation rate of >90% in all daycare facilities and therefore we think the number of tested individuals is sufficient.

Data exclusions

Prospective validation study:
No data were excluded.

Retrospective analysis of the SARS-CoV-2 screening in schools:
SARS-CoV-2 RT-qPCR data of 12 testing laboratories were used for analysis. Only implausible data were excluded: Plausibility was checked with regard to the date of the test, the availability of the assignmend to a school in which the samples were obtained and the availability of a test result. Duplicates were also removed. In total, data of 1,173,704 RT-qPCRs were transmitted by the laboratories. Data of 63,671 RT-qPCRs were excluded because defined criteria for plausibility were not met.

Retrospective analysis of the SARS-CoV-2 screening in daycare facilities:
SARS-CoV-2 RT-qPCR data of two testing laboratories were used for analysis. One private laboratory tested 666 daycare facilities. The other 32 daycare facilities were tested by the laboratory of the Institute of Virology. Since the general data structure for Lolli-testing screening programs was developed by the Institut of Virology in cooperation with the other laboratory, data were 100% valid and complete and no invalid items needed to be removed. Nevertheless, formally the same filter criteria were used as for testing in the schools.

Replication

Prospective validation study:
Individual samples were tested by standardized diagnostic instruments (see methods). In total, 254 independent individuals were tested over a period of more than 4 months. Furthermore, the validation was carried out in Germany and in Mexico in two independent laboratories and by different personnel and with different equipment. There were no findings that could not be reproduced.

Retrospective analysis of the SARS-CoV-2 screening in schools:
The analysis is a retrospective analysis. Each individual was only tested once per test day. Testing was performed twice a week over a period of 13 weeks.

Randomization

Prospective validation study:
Randomization was not applicable because the validation aimed to validate a specimen (Lolli-swab) for the detection of SARS-CoV-2.

Retrospective analysis of the SARS-CoV-2 screening in schools:
Randomization was not applicable. For statistical modelling, the local (district level) 7-day incidence of children aged between 6 and 10 years, the rate of positivity of pool-RT-qPCRs per district and per calendar week and the school social deprivation index (SSDI) were considered as covariates.

Retrospective analysis of the SARS-CoV-2 screening in daycare facilities
Randomization was not applicable. Only the total number of tested pool-and single-RT-qPCRs and positive pool-RT-qPCRs are shown and thus randomization is not relevant.

Blinding

Prospective validation study:
For the validation, the different specimens were obtained with different swab types which could be distiguished from each other. For that

reason, investigators could not blinded. Different specimens were collected from study participiants (Lolli-swabs or Np-/Op-Swabs) which made blinding of study participiants impossible.

Retrospective analysis of the SARS-CoV-2 screening in schools:
Blinding was not applicable. The analysis is a retrosepctive analysis.

Retrospective analysis of the SARS-CoV-2 screening in daycare facilities:
Blinding was not applicable. The analysis is a retrosepctive analysis.

Reporting for specific materials, systems and methods

We require information from authors about some types of materials, experimental systems and methods used in many studies. Here, indicate whether each material, system or method listed is relevant to your study. If you are not sure if a list item applies to your research, read the appropriate section before selecting a response.

Materials & experimental systems

n/a	Involved in the study
x	Antibodies
x	Eukaryotic cell lines
x	Palaeontology and archaeology
x	Animals and other organisms
	x Human research participants
x	Clinical data
x	Dual use research of concern

Methods

n/a	Involved in the study
x	ChIP-seq
x	Flow cytometry
x	MRI-based neuroimaging

Human research participants

Policy information about studies involving human research participants

Population characteristics	Prospective validation study:<br>254 acutely SARS-CoV-2 infected individuals (<14 days after symptom onset or first positive PCR) were recruited for the validation of the Lolli-Method. 122 (48.03%) were female, 130 (51.18%) were male and of 2 (0.79%) the gender was not available. Mean age was 39.7 years, ranging from 0 to 92 years.<br>For determining the specificity, 55 healthy indiviuals were tested. 29 (52.72%) were female, 36 (47.27%) were male. Mean age was 25.05 years, ranging from 0 to 65 years.<br><br>Retrospective analysis of the SARS-CoV-2 screening in schools:<br>According to offcial data, all students attending elementary schools and schools for special needs (n=742,771) participated in the test program. Students had a median age of 8 years (IQR 2 years) with 354,125 (47.69%) being female and 388,646 (52.32%) being male.<br><br>Retrospective analysis of the SARS-CoV-2 screening in daycare facilties:<br>Data on population characteristics were not collected systematically. Based on the known number of tested daycare facilities and the reported pool-sizes, number of tested individuals were estimated 48,149 children within the age of 1 to 6 years and 13,577 staff members.
Recruitment	Prospective validation study:<br>All participants gave their written informed consent before the start of the study. The participants were either study patients of the University Hospital Cologne, Germany or of the test center of the High Specialty Regional Hospital, Villahermosa, Tabasco, Mexico. Study patients of the University Hospital Cologne were visited at home after telephone agreement. Study patients of the test center of the High Specialty Regional Hospital were recruited and consented to participation on site. The participants could only be informed in the respective national language. For this reason, there was a selection bias for citizens of the respective country. However, we do not believe that this has influenced the results.<br><br>Retrospective analysis of the SARS-CoV-2 screening in schools:<br>Testing was a mandatory obligation, imposed by the Ministry for Education of North-Rhine Westphalia. Thus, written informed consent was waived.<br><br>Retrospective analysis of the SARS-CoV-2 screening in daycare facilities<br>Participation in testing was voluntarily for children and staff. The screening program was offered by the Youth Welfare Office to all daycare facilities of Cologne. We estimate the participation of the children and the staff to be over 90% and therefore do not expect a relevant bias of the results.
Ethics oversight	Prospective validation study:<br>The prospective validation study was approved by the Institutional Review Board (IRB) of the Faculty of Medicine and University Hospital of Cologne, Cologne, Germany (number 20-1405) as well as by IRB of the High Specialty Regional, Villahermosa, Mexico (number 0130144).

Retrospective analysis of the SARS-CoV-2 screening in schools:
The retrospective analysis of the SARS-CoV-2 screening in schools by the University Hospital of Colognewas approved by the IRB of the Faculty of Medicine and University Hospital of Cologne, Cologne, Germany (number 21-1358). All analysis was performed in accordance with the Ministry for Education and Ministry of Health of North-Rhine Westphalia.

Retrospective analysis of the SARS-CoV-2 screening in daycare facilities:
For retrospective analysis of the SARS-CoV-2 screening in daycare facilities, the University of Cologne was engaged by the Youth Welfare Office of the city of Cologne and approved by the IRB of the Faculty of Medicine, University Hospital of Cologne, Germany (number 21-1358).

Note that full information on the approval of the study protocol must also be provided in the manuscript.
